# Inhibition of AXL receptor tyrosine kinase increases osteoblast function and bone mass

**DOI:** 10.1038/s41413-026-00554-0

**Published:** 2026-07-06

**Authors:** Mubashir Ahmad, Christoph Kölbl, Irfana Jan, Ann-Kristin Dorn, Burak Özkan, Florian Olde Heuvel, Dilay Yilmaz, Alessa Wagner, Maja Vujic Spasic, Benjamin Thilo Krüger, Sooyeon Lee, Francesco Roselli, Anita Ignatius, Jan Tuckermann

**Affiliations:** 1 Institute of Molecular Endocrinology and Physiology, Ulm University, Ulm, Germany; 2 Institute of Orthopedic Research and Biomechanics, Ulm University Medical Center, Ulm, Germany; 3 Institute of Immunology, Ulm University Medical Center, Ulm, Germany; 4 Department of Neurology, Ulm University, Ulm, Germany; 5 German Center for Neurodegenerative Disease (DZNE)-Ulm, Ulm, Germany; 6 German Center for Child and Adolescent Health (DZKJ), Partner Site Ulm, Ulm, Germany

**Keywords:** Bone, Bone quality and biomechanics

## Abstract

Osteoporosis, a prevalent age-related disease, is characterized by impaired bone formation and an increased risk of fractures. Current anabolic treatments primarily rely on biologics, which are costly and require inconvenient administration. Identifying regulators of osteoblastogenesis that are amenable to small-molecule targeting is essential for developing more accessible therapies. Through an unbiased kinome-wide RNAi screen in primary murine calvarial osteoblasts, we identified the AXL receptor tyrosine kinase (Axl) as a negative regulator of osteoblast differentiation. Axl is most highly expressed in undifferentiated and early differentiated osteoblasts, with a rapid decline in expression during osteoblast maturation. siRNA-mediated knockdown of Axl or pharmacological inhibition with the small molecule BGB324 significantly enhanced osteoblast differentiation and mineralization in vitro. In mice, BGB324 treatment significantly increased bone mass by promoting bone formation. Mechanistically, Axl knockdown or inhibition upregulated interferon-stimulated gene 15 *(Isg15)*, while *Isg15* knockdown impaired osteoblast differentiation and enhanced Erk phosphorylation, leading to increased expression of osteoblast-specific genes. Consistently, double knockdown experiments demonstrated that simultaneous loss of *Axl* with either *Isg15* or *Mapk1*, but not other interferon-related genes, reversed the *Axl* knockdown-induced increase in osteoblast differentiation, reinforcing their mechanistic involvement. Collectively, our study identifies *Axl* as a promising therapeutic target for osteoporosis and other bone-related disorders.

## Introduction

Osteoporosis is a systemic skeletal disorder characterized by reduced bone mass and deterioration of bone microarchitecture, resulting in an increased risk of fractures.^1–3^ As one of the most prevalent age-related diseases worldwide, osteoporosis represents a growing global health burden, particularly in aging populations.^4–6^ With the expansion of the elderly demographic, the incidence of osteoporotic fractures is projected to rise substantially, leading to increased morbidity, mortality, and healthcare expenditures.^7^ This underscores the urgent need for improved therapeutic strategies aimed at preserving skeletal integrity and preventing fractures.^8,9^

Bone homeostasis relies on a tightly regulated balance between osteoblastic bone formation and osteoclastic bone resorption.^10^ Perturbations to this equilibrium, arising from hormonal changes, inflammation, or cellular senescence, contribute to pathological bone loss and fragility.^11–14^ Over the past two decades, significant progress has been made in the development of both anti-resorptive and anabolic agents. Current anti-resorptive therapies, including bisphosphonates and denosumab, effectively reduce osteoclast-mediated bone resorption.^15–17^ In contrast, anabolic treatments such as parathyroid hormone (PTH) analogs and sclerostin inhibitors promote new bone formation.^18^ However, these therapeutic options are limited by suboptimal efficacy, safety concerns, and time-restricted use,^19–21^ highlighting the need to identify novel, osteoanabolic targets that can enhance osteoblast function.

Receptor tyrosine kinases (RTKs) are critical regulators of cell proliferation, survival, and differentiation.^22,23^ Among the TAM family of RTKs, comprising Tyro3, Axl, and Mertk, TYRO3 protein tyrosine kinase 3 *(Tyro3)* and MER proto-oncogene tyrosine kinase *(Mertk)*, have recently emerged as key modulators of bone homeostasis, Notably, inhibition of Mertk has been shown to promote bone formation and represents a potential osteoanabolic approach with broader therapeutic implications in oncology and regenerative medicine,^24^ These findings have prompted further investigation into the role of AXL receptor tyrosine kinase (Axl), another member of the TAM family, in skeletal biology.

Axl is broadly expressed across multiple cell types and plays essential roles in the resolution of inflammation, cell survival, and efferocytosis.^25,26^ Its activation, primarily via binding to its ligand growth arrest-specific 6 (Gas6), triggers downstream signaling through pathways such as PI3K/AKT and MAPK.^27,28^ While Axl has been extensively studied in cancer, immune regulation, and vascular biology,^28–30^ its function in bone biology remains largely unexplored.

Here, we identify *Axl* as a negative regulator of osteoblast differentiation and bone formation. Through kinome-wide RNAi screening in primary calvarial osteoblasts, we demonstrate that genetic silencing or pharmacological inhibition of Axl promotes osteogenesis in vitro and enhances bone mass in vivo. Mechanistically, Axl suppresses osteoblast differentiation via modulation of the MAPK pathway through *Isg15*. Our findings position Axl as a promising therapeutic target for osteoporosis and other bone-related disorders.

## Results

### *Axl* acts as a negative regulator of osteoblast differentiation and mineralization

**Fig. 1:**
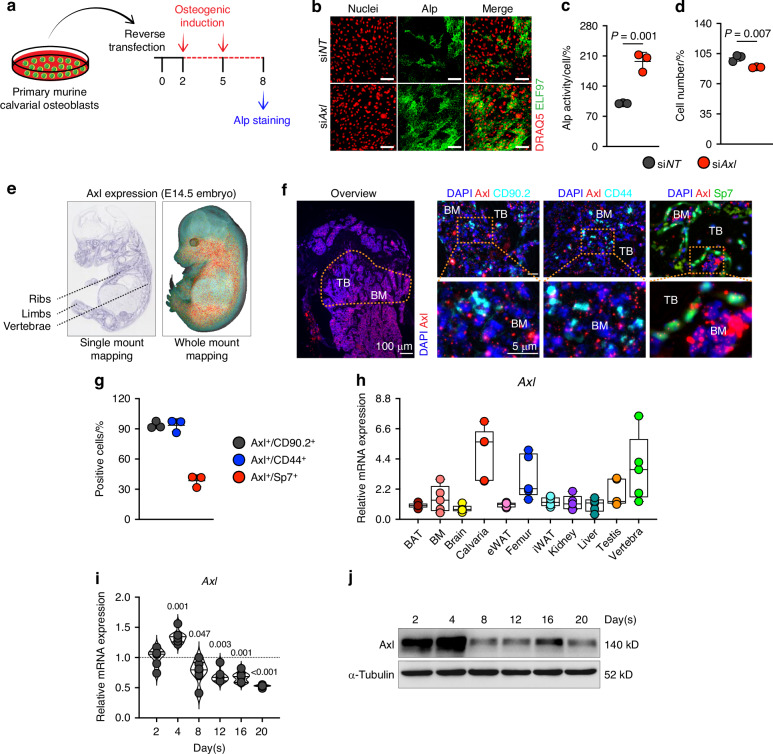
Axl is identified as a negative regulator of osteoblast differentiation. **a** Schematic overview of an RNAi-based kinome screen targeting 719 kinases in primary murine calvarial osteoblasts to identify regulators of osteoblast differentiation, as previously described.^31^ **b** Representative fluorescence images of primary murine calvarial osteoblasts transfected with non-targeting siRNA (si*NT*) or *Axl*-specific siRNA (si*Axl*) at day 8 post-transfection, showing nuclear staining with DRAQ5 (red) and Alp activity with ELF 97 (green). Scale bar, 100 µm. **c**, **d** Quantification of cellular Alp activity and cell numbers in primary murine calvarial osteoblasts transfected with si*NT* or si*Axl* at day 8 post-transfection (*n* = 3). **e** In situ hybridization data from the EMAGE database^36^ demonstrating *Axl* mRNA expression at embryonic day 14.5 (E14.5), with signal localized to ribs, limbs, and vertebrae. **f** Single-molecule mRNA in situ hybridization (RNAscope) combined with immunofluorescence staining of CD90.2, CD44, and Sp7 on femur sections from 12-week-old male mice. **g** Quantification of the fraction of *Axl*-expressing cells within the CD90.2-, CD44-, and Sp7-positive cell populations (TB trabecular bone, BM bone marrow) (*n* = 3). **h** qPCR analysis of *Axl* mRNA expression in various tissues from 13-week-old C57BL/6 male mice, including brown adipose tissue (BAT), bone marrow (BM), brain, calvaria, epididymal white adipose tissue (eWAT), femur, inguinal white adipose tissue (iWAT), kidney, liver, testis, and vertebrae (*n* = 3-5). **i** Temporal analysis of *Axl* mRNA expression during osteoblast differentiation in primary murine calvarial osteoblasts, assessed by qRT-PCR (*n* = 6). **j** Western blot showing Axl protein expression during osteoblast differentiation, with tubulin used as a loading control. Data are presented as aligned dot plots showing the mean and standard deviation. Statistical comparisons between time points were performed using one-way ANOVA followed by Tukey’s post hoc test

To identify kinases involved in the regulation of osteoblast differentiation, we performed an RNA interference (RNAi) screen targeting the mouse kinome in primary calvarial osteoblasts, as previously described.^31^ Among the candidates identified as negative regulators, *Axl* emerged as a potential suppressor of osteoblast differentiation.^31^ siRNA-mediated knockdown of *Axl* markedly enhanced alkaline phosphatase (Alp) activity, as demonstrated by ELF staining in primary murine calvarial osteoblasts (Fig. 1a–c), indicating that *Axl* functions as a repressor of early osteoblast differentiation, despite a concomitant reduction in cell number (Fig. 1d). *Axl* is known to be predominantly expressed in the lungs, spleen, kidneys, and liver.^32–35^ To assess whether *Axl* is also expressed in developing skeletal tissue, we explored the EMAGE gene expression database,^36^ which revealed that *Axl* mRNA is expressed at sites of skeletal development by embryonic day 14.5 (E14.5), with prominent expression in the ribs, limbs, and vertebrae (Fig. 1e). To further define the cellular localization of *Axl* within the adult bone microenvironment, we performed single-molecule mRNA in situ hybridization (RNAscope) on femur sections from 12-week-old male mice. Notably, *Axl* mRNA expression was not restricted to the osteoblast lineage but was broadly distributed across the bone marrow compartment, indicating expression in multiple cell types, including hematopoietic and other non-mesenchymal populations. Combined RNAscope detection of *Axl* transcripts with immunofluorescence staining for osteoblast-lineage and stromal markers demonstrated that *Axl* mRNA is expressed in a high fraction (~93%) of CD90.2- and CD44-positive mesenchymal stromal cells within bone tissue.^37–39^ In contrast, the fraction of *Axl*-expressing cells among osteoblast-committed Sp7-positive cells was lower (~38%), suggesting a decline in *Axl* expression with the onset of osteoblast differentiation (Fig. 1f, g). In adult mice, *Axl* expression was detected in bone tissues including the calvaria, femur and vertebrae, with lower expression levels in brown adipose tissue (BAT), brain and epididymal white adipose tissue (eWAT) (Fig. 1h). Temporal analysis of *Axl* expression during osteoblast differentiation in primary murine calvarial osteoblasts showed that *Axl* mRNA and protein levels were elevated at early stages of differentiation (day 2 and 4), but progressively declined as differentiation proceeded until day 20 (Fig. 1i, j). To further confirm proper osteogenic differentiation in our primary calvarial osteoblast cultures, we additionally quantified the expression of key osteoblast differentiation markers. qPCR analysis revealed a significant increase in alkaline phosphatase *(Alpl)* and bone gamma-carboxyglutamate protein *(Bglap)* mRNA levels over the course of differentiation, supporting that the differentiation protocol robustly induced osteogenic commitment (Fig. S1a–c). Notably, we also assessed the expression of *Gas6*, the principal ligand of Axl, during osteoblast differentiation. Interestingly, *Gas6* mRNA levels progressively increased over the course of differentiation, in contrast to the declining expression of *Axl* (Fig. S1a, d). This pattern suggests that *Gas6* may act in an autocrine or paracrine manner during osteoblast maturation, potentially contributing to the regulation of osteoblast and osteoclast lineages.

**Fig. 2:**
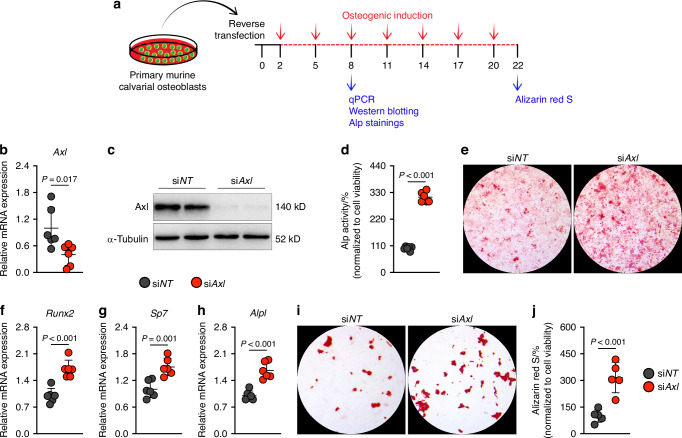
*Axl* knockdown promotes osteoblast differentiation and mineralization in primary murine calvarial osteoblasts. **a** Schematic overview of siRNA-mediated knockdown of *Axl* in primary murine calvarial osteoblasts. **b** *Axl* mRNA expression quantified by qRT-PCR at day 8 post-transfection (*n* = 6). **c** Axl protein levels assessed by western blotting at day 8 post-transfection (*n* = 2). **d**, **e** Quantitative (*n* = 6) and qualitative (*n* = 4) Alp staining in cells transfected with si*NT* or si*Axl* at day 8 post-transfection. **f**–**h** Expression of osteoblast-specific marker genes *(Runx2, Sp7*, and *Alpl)* quantified by qRT-PCR in si*NT*- or si*Axl*-transfected cells at day 8 post-transfection (*n* = 6). **i**, **j** Representative images and quantification of Alizarin Red S (ARS) staining in si*NT*- or si*Axl*-transfected osteoblasts at day 22 post-transfection (*n* = 5). Data are presented as scatter dot plots with mean and standard deviation. Statistical significance between two groups was determined using one-way ANOVA followed by Tukey’s post hoc test

To further validate *Axl* as a negative regulator of osteoblast differentiation, we performed siRNA-mediated knockdown in primary murine calvarial osteoblasts and assessed their differentiation over 20-day period (Fig. 2a). Efficient silencing of *Axl* at both the mRNA and protein levels was confirmed (Fig. 2b, c), without affecting the expression of other TAM receptor family members, including TYRO3 protein tyrosine kinase 3 *(Tyro3)* and MER proto-oncogene tyrosine kinase *(Mertk*) (Fig. S2a–c). *Axl* knockdown significantly increased Alp activity, as assessed by naphthol AS-BI/fast red violet LB staining and a colorimetric Alp assay (Fig. 2d, e). This was accompanied by upregulation of key osteogenic markers, including runt-related transcription factor 2 *(Runx2)*, Sp7 transcription factor 7 *(Sp7)*, and *Alpl* at day 6 of differentiation (Fig. 2f–h). Moreover, enhanced matrix mineralization was observed following *Axl* knockdown, as demonstrated by Alizarin Red S staining at day 20 of differentiation (Fig. 2i, j).

**Fig. 3:**
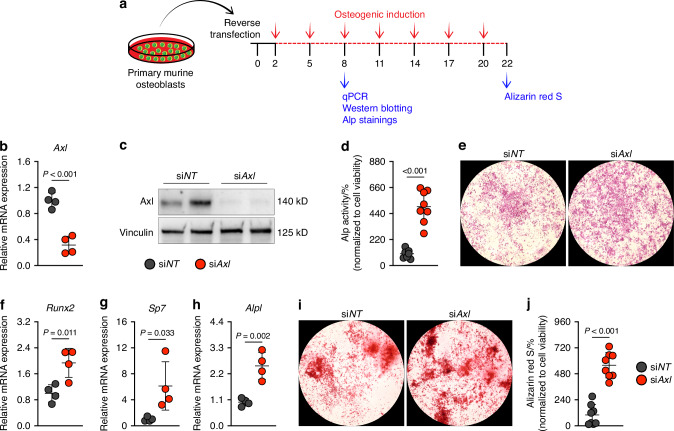
*Axl* knockdown enhances osteoblast differentiation and mineralization in primary murine osteoblasts isolated from long bones. **a** Schematic representation of siRNA-mediated knockdown of *Axl* in primary murine osteoblasts. **b** *Axl* mRNA expression measured by qRT-PCR at day 8 post-transfection (*n* = 4). **c** Axl protein levels assessed by western blotting at day 8 post-transfection (*n* = 2). **d**, **e** Quantitative (*n* = 8) and qualitative (*n* = 4) Alp staining in cells transfected with si*NT* or si*Axl* at day 8 post-transfection. **f**–**h** Expression of osteoblast-specific marker genes *(Runx2, Sp7*, and *Alpl)* quantified by qRT-PCR in si*NT*- or si*Axl*-transfected cells at day 8 post-transfection (*n* = 4). **i**, **j** Representative images and quantification of ARS staining in si*NT*- or si*Axl*-transfected osteoblasts at day 22 post-transfection (*n* = 8). Data are presented as scatter dot plots with mean and standard deviation. Statistical significance between two groups was determined using one-way ANOVA followed by Tukey’s post hoc test

To determine whether this regulatory effect of *Axl* is limited to calvarial osteoblasts or extends to osteoblasts derived from long bones, we performed a similar knockdown in primary murine osteoblasts isolated from the femur and tibia (Fig. 3a). Consistent with the findings in calvarial cells, *Axl* silencing led to increased Alp activity (Fig. 3b–e), elevated expression of osteoblast marker genes *(Runx2, Sp7, and Alpl)* (Fig. 3f–h), and enhanced mineral deposition (Fig. 3i, j). In addition, expression levels of the related TAM receptors *Tyro3* and *Mertk* remained unchanged following *Axl* siRNA knockdown in long-bone-derived osteoblasts (Fig. 3a–c), further supporting the specificity of Axl’s role within this receptor family in regulating osteoblast differentiation. Together, these findings establish *Axl* as a negative regulator of osteoblast differentiation and matrix mineralization.

### Pharmacological inhibition of Axl enhances osteoblast differentiation and bone mass

**Fig. 4:**
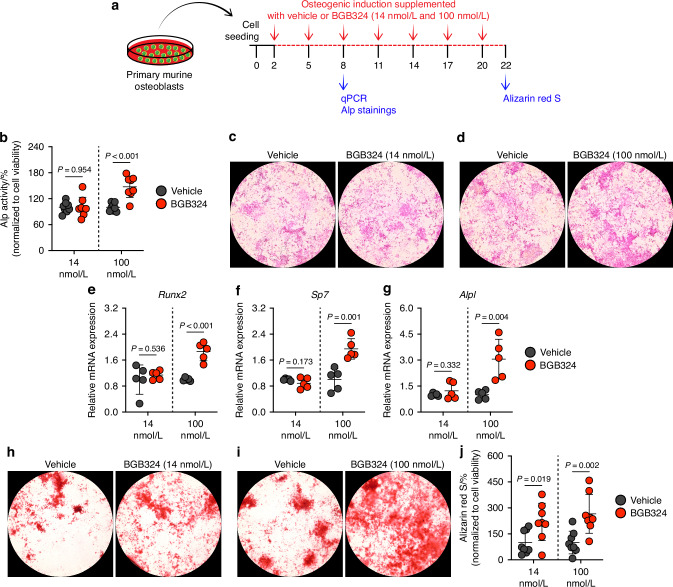
Pharmacological inhibition of Axl with BGB324 in vitro promotes osteoblast differentiation and mineralization in primary murine osteoblasts. **a** Schematic overview of vehicle (DMSO) or BGB324 (14 nmol/L or 100 nmol/L) treatment in primary murine osteoblasts. **b**–**d** Quantitative (*n* = 8) and qualitative (*n* = 4) Alp staining in cells treated with vehicle or BGB324 at day 8 post-seeding. **e**–**g** Expression of osteoblast-specific marker genes *(Runx2, Sp7*, and *Alpl)* measured by qRT-PCR in vehicle- or BGB324-treated cells at day 8 post-seeding (*n* = 5). **h**–**j** Representative images and quantification of ARS staining in vehicle- or BGB324-treated osteoblasts at day 22 post-seeding (*n* = 8). Data are presented as scatter dot plots with mean and standard deviation. Statistical significance between two groups was determined using one-way ANOVA followed by Tukey’s post hoc test

BGB324 is a selective small-molecule inhibitor of Axl that has demonstrated promising efficacy in cancer models^40–43^ and is currently under evaluation in clinical trials.^44^ Based on these findings, we hypothesized that pharmacological inhibition of Axl may enhance osteoblast differentiation and increase bone mass. To test this, we treated primary murine osteoblasts isolated from long bones and calvariae with two concentrations of BGB324 (14 nmol/L, the reported IC_50_ and 100 nmol/L), as previously described^45^ (Fig. 4a, Fig. S4a). Treatment with 14 nmol/L BGB324 did not significantly affect Alp activity (Fig. 4b, c), or the expression of osteoblast marker genes *(Runx2, Sp7, Alpl)* in osteoblasts derived from long bones (Fig. 4e–g). However, in calvarial osteoblasts, 14 nmol/L BGB324 significantly increased Alp activity (Fig. S4b, c) without affecting cell numbers (Fig. S4b, d), and upregulated *Runx2* expression (Fig. S4e–g). No significant change in matrix mineralization was observed in long-bone-derived osteoblasts at this dose (Fig. 4h, j). In contrast, treatment with 100 nmol/L BGB324 led to a marked increase in both quantitative and qualitative Alp activity (Fig. 4b, d), upregulation of osteoblast marker gene expression (*Runx2, Sp7*, and *Alpl*) (Fig. 4e–g), and enhanced matrix mineralization (Fig. 4i, j) in long-bone-derived osteoblasts. Thus, pharmacological inhibition with different sensitivities enhanced osteoblast differentiation of primary cells isolated from calvaria and long bones.

**Fig. 5:**
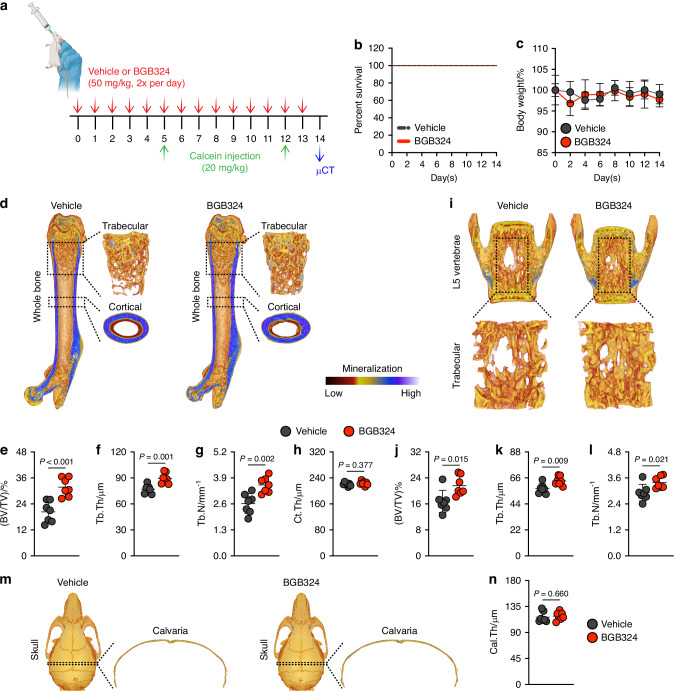
Pharmacological inhibition of Axl with BGB324 increases bone mass in vivo. **a** Experimental design for treatment of 11-week-old female BALB/c mice with vehicle (0.5% (w/v) methylcellulose and 0.1% (w/v) Tween 80 in water) or BGB324 (50 mg/kg, administered by oral gavage twice daily, 8 h apart for two weeks). **b**, **c** Percent survival and body weight of mice treated with vehicle or BGB324 (*n* = 7). **d** Representative microcomputed tomography (µCT) images of whole femur, trabecular bone, and cortical bone from vehicle- or BGB324-treated mice. **e**–**h** Quantification of femoral bone parameters: bone volume fraction (BV/TV, %), trabecular thickness (Tb.Th, µm), trabecular number (Tb.N, mm^−^^1^), and cortical thickness (Ct.Th, µm) (*n* = 7). **i** Representative µCT images of whole vertebra and trabecular bone from vehicle- or BGB324-treated mice. **j**–**l** Quantification of vertebral trabecular bone parameters: BV/TV (%), Tb.Th (µm), and Tb.N (mm^−^^1^) (*n* = 7). **m** Representative µCT images of whole skull and calvarial bone from vehicle- or BGB324-treated mice. **n** Quantification of calvarial thickness (Ca.Th, µm) (*n* = 7). Data are presented as scatter dot plots with mean and standard deviation. Statistical significance between two groups was determined using one-way ANOVA followed by Tukey’s post hoc test

To determine whether BGB324 treatment impacts bone mass in vivo, 12-week-old female BALB/c mice were treated with BGB324 or vehicle twice per day for two weeks (Fig. 5a). BGB324 was well tolerated, with no evidence of toxicity as assessed by survival and body weight monitoring (Fig. 5b, c). Blood cell counts showed a reduction in white blood cells, specifically lymphocytes and granulocytes, whereas monocytes and other cell populations were largely unaffected (Table 1). Microcomputed tomography (µCT) analysis of femurs revealed a significant increase in trabecular bone volume fraction (BV/TV), trabecular thickness (Tb.Th), and trabecular number (Tb.N), while cortical thickness (Ct.Th) remained unchanged (Fig. 5d–h). Similar trends were observed in the axial skeleton, where µCT analysis of L5 vertebrae demonstrated increased BV/TV, due to elevated Tb.Th and Tb.N (Fig. 5i–l). To further assess the effects of BGB324 on cranial bone, µCT analysis of the calvaria was performed. Consistent with the lack of cortical bone alterations observed in long bones, no measurable structural changes in calvarial bone architecture were detected following two weeks of treatment (Fig. 5m, n).

**Table 1:** Hematological parameters in mice treated with BGB324

Parameter	Vehicle (*n* = 6)	BGB324 (*n* = 5)	*P*-Value
WBCs/(K/µL)	13.75 ± 0.83	4.52 ± 1.03	<0.001
RBCs/(M/µL)	8.94 ± 0.22	8.33 ± 0.66	0.400
HBG/(g/dL)	18.60 ± 0.50	15.93 ± 1.69	0.161
HCT/%	50.08 ± 0.99	43.38 ± 4.36	0.164
MCV/fL	56.17 ± 0.40	54.33 ± 0.42	0.010
MCH/pg	20.82 ± 0.19	21.18 ± 0.68	0.615
MCHC/(g/dL)	37.12 ± 0.42	39.05 ± 1.44	0.224
PLT/(K/µL)	281.83 ± 122.27	193.83 ± 67.19	0.542
MPV/fL	6.88 ± 0.37	7.20 ± 0.23	0.484
RDW/%	13.05 ± 0.08	12.92 ± 0.12	0.376
% LYM	79.48 ± 1.20	77.46 ± 2.51	0.490
% MON	4.32 ± 0.50	4.88 ± 0.43	0.437
% GRA	16.03 ± 0.70	17.60 ± 2.12	0.501
# LYM/(K/µL)	10.30 ± 0.98	5.32 ± 1.37	0.018
# MON/(K/µL)	0.50 ± 0.09	0.36 ± 0.15	0.455
# GRA	2.25 ± 0.24	0.96 ± 0.16	0.002

Data are shown as mean ± standard deviation. Statistical significance between two groups was determined using one-way ANOVA followed by Tukey’s post hoc test

To evaluate whether these effects persist during prolonged inhibition of Axl, BGB324 treatment was extended to 28 days in an independent cohort of 9-week-old female BALB/c mice (Fig. S5a). BGB324 remained well tolerated, with no evidence of toxicity based on survival and body weight monitoring (Fig. S5b, c). µCT analysis of femurs again revealed a significant increase in trabecular BV/TV, Tb.Th, and Tb.N, while Ct.Th was also significantly increased (Fig. S5d–h). Similar trends were observed in the axial skeleton, where µCT analysis of L5 vertebrae demonstrated increased BV/TV, driven primarily by elevated Tb.N (Fig. S5i–l). Collectively, these results demonstrate that pharmacological inhibition of Axl with BGB324 promotes osteoblast differentiation in vitro and enhances trabecular bone formation in vivo, without producing detectable changes in calvarial bone architecture under these treatment conditions.

### Inhibition of Axl with BGB324 enhances markers of bone formation and persistently increases osteocyte numbers

**Fig. 6:**
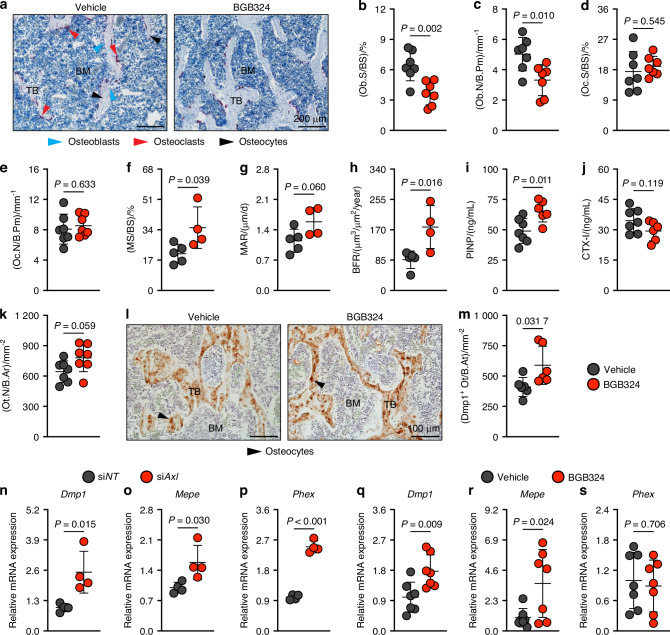
Pharmacological inhibition of Axl with BGB324 increases bone mass in vivo by enhancing bone formation. **a** Representative tartrate-resistant acid phosphatase (TRAP) staining for femoral trabecular bone sections from vehicle- or BGB324-treated mice, showing osteoblasts (blue arrows), osteoclasts (red arrows), and osteocytes (black arrows) (scale bar: 200 µm). **b**–**e** Quantification of histomorphometric parameters from femoral trabecular bone: osteoblast surface per bone surface (Ob.S/BS, %), osteoblast number per bone perimeter (Ob.N/B.Pm, mm^−^^1^), osteoclast surface per bone surface (Oc.S/BS, %), and osteoclast number per bone perimeter (Oc.N/B.Pm, mm^−^^1^) (*n* = 7). **f**–**h** Dynamic bone histomorphometry of femurs from vehicle- or BGB324-treated mice showing mineralizing surface per bone surface (MS/BS, %), mineral apposition rate (MAR, µm/d), and bone formation rate (BFR, µm^3^/µm^2^/year) (*n* = 4–5). **i**, **j** Plasma levels of bone formation and resorption markers: procollagen type I N-terminal propeptide (PINP, ng/mL) and C-terminal telopeptide of type I collagen (CTX-I, ng/mL) (*n* = 7). **k** Osteocyte number per bone area (Ot.N/B.Ar, mm^−^^2^) in femurs from vehicle- or BGB324-treated mice (*n* = 7). **l** Representative images of Dmp1 staining of femoral trabecular bone sections from vehicle- or BGB324-treated mice. **m** Quantification of Dmp1^+^ osteocytes per bone area (Dmp1^+^ Ot/B.Ar, mm^−^^2^) in femurs from vehicle- or BGB324-treated mice (*n* = 6). **n**–**p** Expression of osteocyte-specific marker genes *(Dmp1, Mepe*, and *Phex)* quantified by qRT-PCR in primary murine osteoblasts transfected with si*NT* or si*Axl* at day 8 post-transfection (*n* = 4). **q**–**s** Expression of *Dmp1, Mepe*, and *Phex* in radii/ulnae from vehicle- or BGB324-treated mice measured by qRT-PCR (*n* = 7). Data are presented as scatter dot plots with mean and standard deviation. Statistical significance between two groups was determined using one-way ANOVA followed by Tukey’s post hoc test

To delineate the cellular mechanisms underlying the anabolic effects of Axl inhibition, we performed static and dynamic bone histomorphometry. At the 14-day time point, the increase in trabecular bone mass detected by µCT was not accompanied by an increase in osteoblast numbers. Instead, BGB324 treatment resulted in a significant reduction in osteoblast surface per bone surface (Ob.S/BS) and osteoblast number per bone perimeter (Ob.N/B.Pm) (Fig. 6a–c), while osteoclast surface per bone surface (Oc.S/BS) and osteoclast number per bone perimeter (Oc.N/B.Pm) remained unchanged (Fig. 6a, d, e). Despite the reduced osteoblast numbers, dynamic histomorphometric analysis revealed a significant increase in mineralizing surface per bone surface (MS/BS), mineral apposition rate (MAR), and bone formation rate (BFR), indicating enhanced bone formation activity (Fig. 6f–h). Consistent with these findings, plasma biochemical analyses showed elevated levels of the bone formation marker N-terminal propeptide of type I procollagen (PINP), while the bone resorption marker C-terminal telopeptides of type I collagen (CTX-I) remained unchanged (Fig. 6i, j). Given the apparent paradox of reduced osteoblast numbers despite increased bone formation, we hypothesized that BGB324 treatment may enhance early osteoblast activity followed by their maturation into osteocytes, thereby contributing to a reduction in osteoblast numbers. In line with this, osteocyte number per bone area (Ot.N/B.Ar) was significantly increased following BGB324 treatment (Fig. 6a, k). Immunohistochemical analysis of paraffin sections further demonstrated a significant increase in Dmp1^+^ osteocytes per bone area (Dmp1^+^ Ot/B.Ar), suggesting an enhanced osteoblast maturation and the accumulation of osteocytes (Fig. 6l, m). In vitro, siRNA-mediated knockdown of *Axl* similarly promoted the osteocyte differentiation program, as evidenced by upregulation of osteocyte marker genes including dentin matrix protein 1 *(Dmp1)*, matrix extracellular phosphoglycoprotein *(Mepe),* and phosphate-regulating endopeptidase homolog, X-linked *(Phex)* (Fig. 6n–p). Consistent with these findings, gene expression analysis of radii/ulnae from BGB324-treated mice revealed increased expression of *Dmp1* and *Mepe* (Fig. 6q–s).

To determine whether these effects persist during prolonged Axl inhibition, we performed additional histomorphometric analyses after 28 days of BGB324 treatment. At this later time point, static histomorphometry showed no significant differences in osteoblast or osteoclast parameters compared with vehicle-treated controls, whereas osteocyte numbers remained elevated (Fig. S6a–f). In contrast to the 14-day treatment, dynamic histomorphometric parameters, including MS/BS, MAR, and BFR, were no longer significantly altered (Fig. S6g–i). Consistent with these findings, circulating levels of PINP and CTX-I were also unchanged after 28 days of treatment (Fig. S6j, k). Together, these results suggest that short-term Axl inhibition stimulates bone formation and increases trabecular bone mass through enhanced early osteoblast activity, followed by a possible maturation of osteoblasts into osteocytes. After longer treatment, osteoblast numbers and bone formation rate return to baseline, while osteocyte numbers remain elevated, suggesting stabilization and maturation of the newly formed bone without evidence of increased bone resorption or excessive bone turnover.

### *Axl* inhibits osteoblast differentiation by downregulating *Isg15* expression

**Fig. 7:**
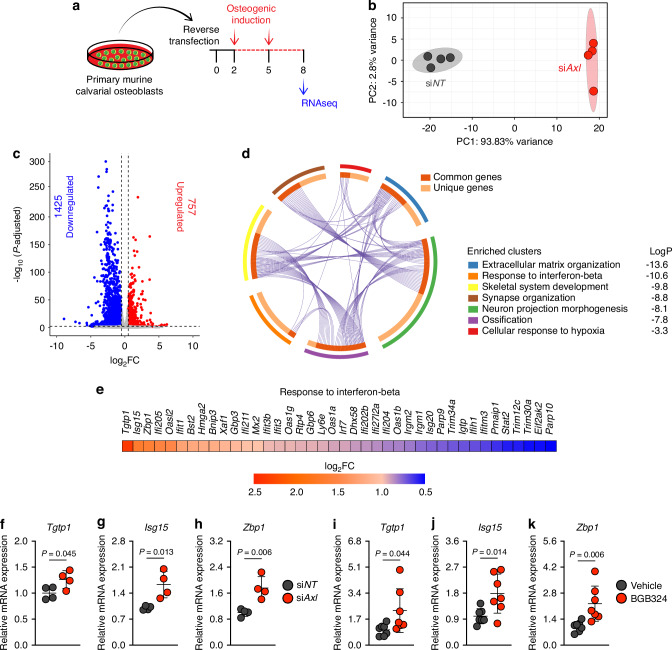
Axl suppresses osteoblast differentiation by modulating *Isg15* expression. **a** Schematic overview of the experimental design. **b** Principal component analysis (PCA) of transcriptomic profiles from RNA sequencing (RNAseq) of primary murine calvarial osteoblasts transfected with si*NT* or si*Axl* (*n* = 4). Samples with similar expression profiles are clustered and color-coded. **c** Volcano plot showing differentially expressed genes (DEGs) between si*NT-* and si*Axl*-transfected osteoblasts. The x-axis represents log_2_ fold change (log_2_FC) and the y-axis represents -log_10_ (adjusted *p*-value). Red and blue dots indicate significantly upregulated and downregulated genes, respectively (log_2_FC ≥ 0.5 or ≤−0.5; FDR < 0.01), gray dots denote non-significant genes. **d** Metascape analysis of DEGs reveals seven highly enriched biological pathways. The circular plot displays the overlap and specificity of genes among these pathways. **e** Genes associated with the interferon-beta response pathway, ranked by log_2_FC from RNAseq analysis. **f**–**h** qRT-PCR validation of selected interferon-stimulated genes *(Tgtp1, Isg15*, and *Zbp1)* in *siNT-* or si*Axl-*transfected primary murine osteoblasts at day 8 post-transfection (*n* = 4). **i**–**k** mRNA expression of *Tgtp1, Isg15*, and *Zbp1* measured by qRT-PCR in radii/ulnae from vehicle- or BGB324-treated mice (*n* = 7). Data are presented as scatter dot plots with mean and standard deviation. Statistical significance between two groups was determined using one-way ANOVA followed by Tukey’s post hoc test

To elucidate the molecular mechanisms by which *Axl* regulates osteoblast differentiation, we performed RNAseq following siRNA-mediated knockdown of *Axl* in primary murine calvarial osteoblasts (Fig. 7a). Principal component analysis (PCA) revealed distinct transcriptional profiles between non-targeting control (si*NT*) and *Axl*-depleted cells, indicating substantial transcriptomic changes (Fig. 7b). Differential gene expression analysis identified 2 182 altered genes, with 757 upregulated and 1 425 downregulated upon *Axl* knockdown (Fig. 7c). Gene ontology enrichment analysis using Metascape highlighted several biological processes, including extracellular matrix organization, response to interferon-beta, skeletal system development (Fig. 7d). While the enrichment of pathways related to extracellular matrix and skeletal development aligned with the observed increase in osteoblast differentiation and bone formation following *Axl* knockdown and inhibition, we also discovered the enrichment of interferon-beta response genes. This interferon-beta regulatory axis is well-documented in macrophages, where Axl functions to suppress interferon-beta signaling and limit antiviral gene expression.^46^ However, such a mechanism has not been previously described in osteoblasts. Focusing on this pathway, we identified the top upregulated genes by log_2_ fold change, including T-cell-specific GTPase 1 *(Tgtp1)*, interferon-stimulated gene 15 *(Isg15)*, and Z-DNA binding protein 1 *(Zbp1)* (Fig. 7e). qPCR validation confirmed that knockdown of *Axl* led to significant upregulation of *Tgtp1, Isg15, and Zbp1* in vitro (Fig. 7f–h). Consistently, analysis of radii/ulnae from BGB324-treated mice showed increased expression of these genes in vivo (Fig. 7i–k).

**Fig. 8:**
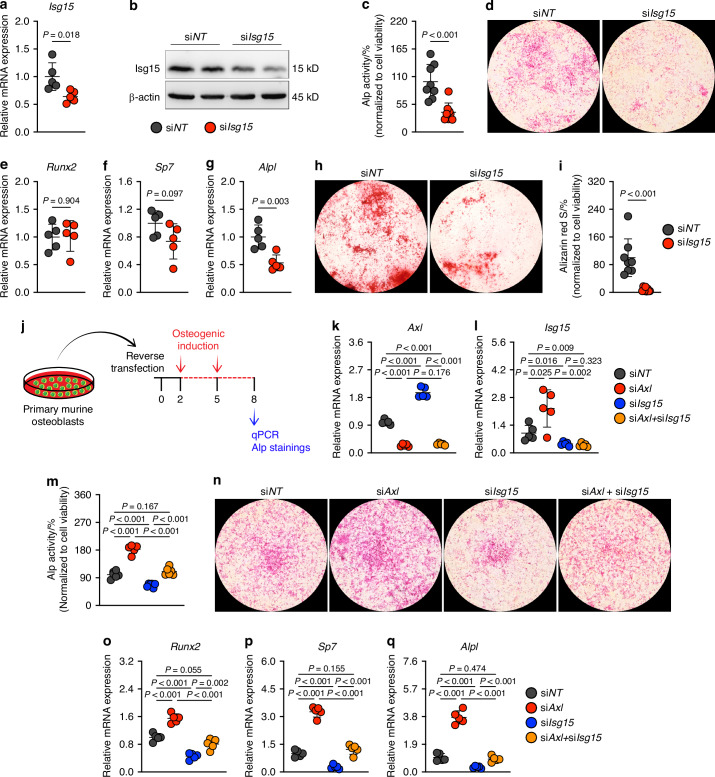
Isg15 knockdown decreases osteoblast differentiation and mineralization in primary murine osteoblasts. **a** *Isg15* mRNA expression measured by qRT-PCR at day 8 post-transfection (*n* = 5). **b** Isg15 protein levels assessed by western blotting at day 8 post-transfection (*n* = 2). **c**, **d** Quantitative (*n* = 8) and qualitative (*n* = 8) Alp staining in cells transfected with si*NT* or si*Isg15* at day 8 post-transfection. **e**–**g** Expression of osteoblast-specific marker genes *(Runx2, Sp7*, and *Alpl)* quantified by qRT-PCR in si*NT*- or si*Isg15*-transfected cells at day 8 post-transfection. **h**, **i** Representative images and quantification of ARS staining in si*NT*- or si*Isg15*-transfected osteoblasts at day 22 post-transfection (*n* = 8). **j** Schematic representation of siRNA-mediated knockdown of *Axl, Isg15*, and co-transfection of *Axl* and *Isg15* in primary murine osteoblasts. **k**, **l** *Axl* and *Isg15* mRNA expression measured by qRT-PCR at day 8 post-transfection (*n* = 5). **m**, **n** Quantitative (*n* = 6) and qualitative (*n* = 6) Alp staining in cells transfected with si*NT*, si*Axl*, si*Isg15*, or co-transfection of si*Axl* and si*Isg15* at day 8 post-transfection. **o**–**q** Expression of osteoblast-specific marker genes *(Runx2, Sp7*, and *Alpl)* quantified by qRT-PCR in cells transfected with si*NT*, si*Axl*, si*Isg15*, or co-transfection of si*Axl* and si*Isg15* at day 8 post-transfection (*n* = 5). Data are presented as scatter dot plots with mean and standard deviation. Statistical significance between two groups was determined using one-way ANOVA followed by Tukey’s post hoc test

To investigate their functional roles, we performed siRNA-mediated knockdown of *Tgtp1, Isg15, and Zbp1* in primary murine osteoblasts and confirmed efficient silencing (Fig. 8a, b, Fig. S7a, b, Fig. S8a, b). Notably, knockdown of *Isg15* resulted in a significant reduction in Alp activity (Fig. 8c, d), decreased expression of the osteogenic marker *Alpl* (Fig. 8e–g), and impaired matrix mineralization (Fig. 8h, i). In contrast, knockdown of *Tgtp1 and Zbp1* reduced Alp activity (Fig. S7c, d, Fig. S8c, d) but did not affect osteogenic gene expression (Fig. S7e–g, Fig. S8e–g).

To further test the mechanistic link between *Axl* and *Isg15*, we performed double knockdown experiments. We first confirmed the efficient deletion of *Axl* and *Isg15* in single and double knockdown conditions (Fig. 8j–l). Simultaneous depletion of *Axl* and *Isg15* reversed the enhanced osteoblast differentiation phenotype observed with *Axl* knockdown alone, as demonstrated by quantitative and qualitative Alp staining (Fig. 8m, n) and by reduced expression of osteoblast markers (*Runx2, Sp7, Alpl*) (Fig. 8o–q). In contrast, double knockdown of *Axl* with *Tgtp1* or *Zbp1* did not reverse the *Axl* knockdown-induced increase in osteoblast differentiation (Fig. S7h–n, Fig. S8h–n).

Together, these findings identify *Isg15* as a key downstream effector of *Axl* signaling. Our data suggest that *Axl* suppresses osteoblast differentiation at least in part by repressing *Isg15*, thereby positioning *Isg15* as a positive regulator of osteoblast maturation and function.

### *Axl* regulates osteoblast function via the Erk-Isg15 signaling axis

**Fig. 9:**
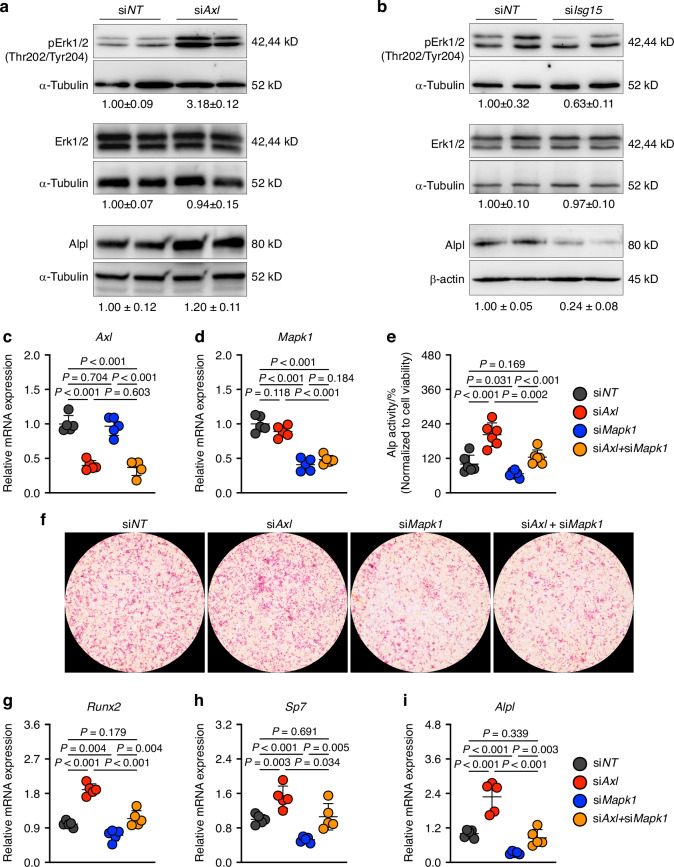
Axl regulates osteoblast differentiation via the MAPK pathway. **a** Western blot analysis of phosphorylated Erk1/2 (pErk1/2, Thr^202^/Tyr^204^), total Erk1/2, and Alpl protein levels in primary murine osteoblasts transfected with si*NT* or si*Axl* at day 8 post-transfection (*n* = 2). **b** Western blots of pErk1/2 (Thr^202^/Tyr^204^), total Erk1/2, and Alpl protein levels in primary murine osteoblasts transfected with si*NT* or si*Isg15* at day 8 post-transfection (*n* = 2). The band intensities were quantified using ImageJ. **c**, **d** *Axl* and *Mapk1* mRNA expression measured by qRT-PCR at day 8 post-transfection (*n* = 5). **e**, **f** Quantitative (*n* = 6) and qualitative (*n* = 6) Alp staining in cells transfected with si*NT*, si*Axl*, si*Mapk1*, or co-transfection of si*Axl* and si*Mapk1* at day 8 post-transfection. **g**–**i** Expression of osteoblast-specific marker genes *(Runx2, Sp7*, and *Alpl)* quantified by qRT-PCR in cells transfected with si*NT*, si*Axl*, si*Mapk1*, or co-transfection of si*Axl* and si*Mapk1* at day 8 post-transfection (*n* = 5). Data are presented as scatter dot plots with mean and standard deviation. Statistical significance between two groups was determined using one-way ANOVA followed by Tukey’s post hoc test

To further delineate the signaling pathway through which *Axl* modulates osteoblast differentiation, we focused on the mitogen-activated protein kinase (MAPK) pathway, as Axl is known to regulate MAPK signaling,^28,47^ and MAPK activity is essential for osteoblast differentiation and bone development.^48,49^ We specifically examined the involvement of extracellular signal-regulated kinase (Erk) signaling. siRNA-mediated knockdown of *Axl* in primary murine osteoblasts resulted in a marked increase in phosphorylated Erk1/2 (pErk1/2) levels, while total Erk1/2 protein levels remained unchanged (Fig. 9a). This increase in Erk1/2 activation was accompanied by an upregulation of Alpl expression (Fig. 9a). Conversely, knockdown of *Isg15* led to reduced Erk1/2 phosphorylation without altering total Erk1/2 levels (Fig. 9b), and was associated with decreased Alpl expression (Fig. 9b), suggesting that *Isg15* is required to sustain Erk1/2 signaling and osteoblast differentiation, positioning it as a downstream mediator of *Axl* function in osteoblasts.

To further test the mechanistic link between Axl and Mapk1, we performed double knockdown experiments targeting *Axl* and *Mapk1*. We confirmed efficient deletion under single and combined knockdown conditions (Fig. 9c, d). Simultaneous loss of *Axl* and *Mapk1* rescued the enhanced osteoblast differentiation phenotype observed with *Axl* knockdown alone, as shown by quantitative and qualitative Alp staining (Fig. 9e, f) and osteoblast marker gene expression (*Runx2, Sp7, Alpl*) (Fig. 9g–i).

**Fig. 10:**
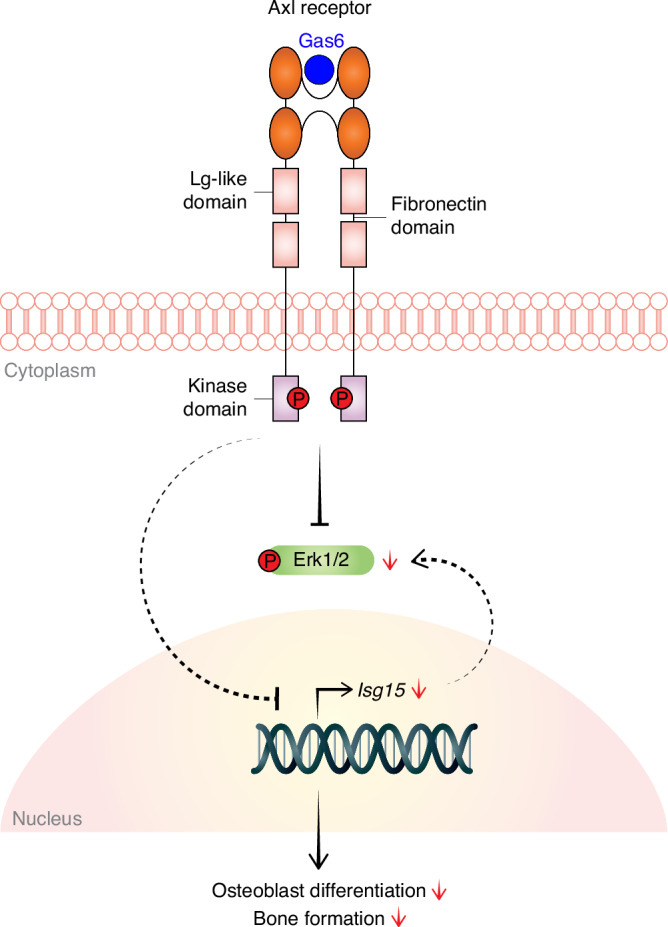
Proposed model. Proposed model illustrating that Axl functions as a negative regulator of osteoblast differentiation by suppressing Erk1/2 phosphorylation and subsequently downregulating *Isg15* expression. However, the possibility that Axl directly regulates *Isg15* independently of Erk1/2, or that *Isg15* contributes to a feedback loop influencing Erk1/2 phosphorylation, cannot be ruled out

Collectively, these findings position Axl as a negative regulator of osteoblast differentiation, functioning through the downregulation of *Isg15*, which in turn suppresses Erk1/2 phosphorylation (Fig. 10). Although our data support an Axl-Isg15-Erk signaling axis in osteoblasts, we cannot exclude the possibility that Axl directly regulates *Isg15* transcription or that Erk1/2 activation influences *Isg15* expression. These alternative mechanisms warrant further investigation in future studies.

## Discussion

In this study, we identify Axl, a receptor tyrosine kinase of the TAM family, as a previously unrecognized negative regulator of osteoblast differentiation and bone formation. Through comprehensive in vitro and in vivo analyses, we demonstrate that genetic ablation or pharmacological inhibition of Axl enhances osteoblast activity, accelerates the osteoblast-to-osteocyte transition, and increases bone mass. Our findings uncover a novel regulatory mechanism involving the Axl-Isg15-Erk axis in osteoblasts, positioning Axl as a promising osteoanabolic target and offering new insights into the molecular network governing osteoblast function and skeletal homeostasis.

Despite multiple therapies available for osteoporosis, there remains a critical need for anabolic agents that can robustly stimulate bone formation without the adverse effects associated with current treatments.^20^ Intermittent-PTH and sclerostin inhibitors, for instance, are constrained by limited treatment duration and safety concerns.^19,20,50,51^ Receptor tyrosine kinases (RTKs), which are key regulators of proliferation, survival, and differentiation, have emerged as important drug targets in cancer and inflammatory diseases.^22,27,28,52^ Axl, extensively studied in cancer biology and immune regulation, plays known roles in survival, proliferation, and immune evasion.^25,26^ While other TAM receptors, such as Tyro3 and Mertk, have been recently implicated in bone homeostasis,^24^ our study extends this paradigm by identifying Axl as a suppressor of osteoblast maturation. Notably, *Axl* knockdown did not induce compensatory expression of *Tyro3* or *Mertk*, supporting distinct and non-redundant roles for TAM family members in bone biology.^53,54^

Our findings are further supported by an RNAi-based kinome screen, which identified *Axl* as a negative regulator of differentiation.^31^ Axl was highly expressed during the early stages of skeletal development and declined with osteoblast maturation, both in vitro and in intact bone. Sp7-committed osteoblast progenitor cells expressed lower levels of *Axl* compared to presumably uncommitted mesenchymal stromal cells. This pattern suggests a temporally restricted and inhibitory role of *Axl*, consistent with other known suppressors of osteogenesis.^31,55^ Loss of *Axl* function significantly enhanced Alp activity, osteogenic gene expression, and matrix mineralization in both cranial and appendicular osteoblasts, indicating a conserved regulatory function across skeletal progenitors.^56–58^

Pharmacological inhibition of Axl using BGB324, a clinically advanced oral inhibitor,^59^ recapitulated the cellular phenotype observed following *Axl* silencing. In vivo, BGB324 administration to adult mice increased trabecular bone mass in long bones and vertebrae, without affecting cortical bone parameters or causing systemic toxicity. Dynamic histomorphometry performed after 14 days of treatment revealed increased BFR and MAR, consistent with a bona fide early anabolic effect.^60^ Interestingly, this occurred despite reduced osteoblast number and surface, suggesting that Axl inhibition enhances osteoblast functionality and/or promotes a more advanced maturation state rather than simply expanding the osteoblast population. Importantly, additional analyses at a later time point refined this interpretation. After 28 days of BGB324 treatment, osteoblast numbers and dynamic bone formation parameters returned to baseline, whereas osteocyte numbers remained elevated. These findings suggest that Axl inhibition induces a transient early phase of increased osteoblast activity and bone formation, followed by stabilization and maturation of the newly formed bone. Notably, this later phase occurred without increased bone resorption or evidence of excessive bone turnover, as circulating levels of PINP and CTX-I remained unchanged. Although these findings strongly support a direct role of Axl within the osteoblast lineage, we cannot exclude the possibility that systemic pharmacological inhibition exerts indirect effects through other Axl-expressing cell types, including immune and endothelial compartments. For example, white blood cells—specifically lymphocytes and granulocytes—were reduced, consistent with the known therapeutic effects of BGB324 in certain leukemia models. The extent to which these hematopoietic changes influence bone formation remains unclear. Thus, while our data using BGB324 treatment aligns with the osteoblast-autonomous effects observed in vitro, indirect systemic contributions to bone formation cannot be excluded. Future studies employing conditional *Axl* deletion models will therefore be essential to dissect cell-type-specific functions. In particular, generating mesenchymal stem cell (MSC)-specific, osteoblast-specific, and osteocyte-specific *Axl* knockout mice would provide critical insight into the precise and lineage-restricted roles of *Axl* in skeletal homeostasis.

To further investigate this paradox of diminished osteoblast numbers in vivo despite enhanced osteoblast differentiation in vitro following Axl inhibition, we examined the osteoblast-to-osteocyte transition, a critical step for matrix embedding and long-term bone maintenance.^61,62^ The early phase of osteoblast differentiation is likely the key stage at which Axl exerts its regulatory influence. In osteoblast cultures, knockdown of *Axl* increased the expression of osteocyte-associated genes, including *Dmp1*, *Mepe,* and *Phex*. Consistent with these findings, our in vivo histomorphometric analyses revealed increased osteocyte numbers following Axl inhibition, suggesting accelerated maturation of osteoblasts into the osteocyte lineage. In agreement, immunostaining for Dmp1 was increased after BGB324 treatment. Similarly, elevated expression of *Dmp1*, *Mepe*, and *Phex* in osteoblast cultures following *Axl* deletion further supports enhanced progression toward an osteocyte fate. The temporal pattern observed in our study, an early increase in bone formation followed by normalization of osteoblast numbers and bone formation parameters at later stages, further supports this interpretation. However, due to the lack of lineage-tracing studies, definitive evidence of osteoblast-to-osteocyte transition in vivo remains to be established. Together, these findings support a model in which Axl constrains terminal differentiation and maintains osteoblasts in a more progenitor-like state, consistent with its role in preserving stem-like features and limiting maturation in other tissues.^63^

At the molecular level, transcriptomic profiling identified *Isg15* as a downstream effector of *Axl* in osteoblasts. *Isg15* is an interferon-stimulated ubiquitin-like modifier involved in antiviral responses, immune regulation, and differentiation.^64–66^ *Axl* knockdown upregulated *Isg15* expression, while *Isg15* silencing impaired Alp activity, osteogenic gene expression, and matrix mineralization, indicating its role as a positive regulator of osteoblast differentiation. Importantly, experiments with combined knockdown of both *Axl* and *Isg15* revealed that the enhanced osteoblast differentiation observed upon *Axl* depletion is dependent on *Isg15*, whereas knockdown of other interferon-stimulated genes, such as *Tgtp1* and *Zbp1*, does not rescue the phenotype. This finding provides direct mechanistic evidence that *Axl* exerts its inhibitory effects on osteoblast differentiation specifically through *Isg15*. These findings also align with previous reports linking *Isg15* to bone metabolism.^67^

Mechanistically, *Axl* silencing increased phosphorylation of Erk1/2, a key branch of the MAPK signaling pathway essential for osteoblast differentiation.^48,49^ Notably, *Isg15* knockdown reduced Erk1/2 phosphorylation, suggesting that *Isg15* functions downstream of *Axl* to sustain Erk signaling and promote osteogenic commitment. Consistent with this model, prior studies have shown that Axl inhibition upregulates *Isg15* expression in various cell types,^68,69^ whereas Axl overexpression suppresses it.^70^ Moreover, ISG15 has been reported to activate the ERK1/2 pathway and promote self-renewal, migration, and tumorigenicity,^71,72^ supporting the existence of an Axl-Isg15-Erk signaling axis. To further substantiate the involvement of MAPK signaling downstream of Axl, we performed a combined knockdown of *Axl* and *Mapk1*. Remarkably, dual silencing abolished the enhanced osteoblast differentiation induced by *Axl* knockdown alone, as demonstrated by reductions in Alp activity and osteoblast marker gene expression (*Runx2, Sp7, Alpl*). This genetic interaction places *Mapk1* as a necessary mediator of the pro-osteogenic effects observed upon *Axl* depletion. These findings indicate that *Axl* restrains osteoblast differentiation at least in part by suppressing Erk1/2 activation, and that intact MAPK signaling is required for the differentiation-promoting phenotype triggered by *Axl* loss. While our findings substantiate this axis, we cannot exclude the possibility that Axl directly regulates *Isg15* transcription, or that a bidirectional interaction exists between Erk signaling and *Isg15* expression. Further proteomic and interactome studies will be necessary to delineate this mechanism in greater detail.

Our transcriptomic data further revealed upregulation of interferon-stimulated genes such as *Tgtp1* and *Zbp1* following *Axl* depletion. The suppression of type I interferon responses by Axl is well established in immune cells.^73,74^ We propose a similar axis in osteoblasts, where Axl inhibits IFN-ß signaling and its downstream gene network. Since interferons exert context-dependent effects on bone remodeling,^75^ future studies should clarify the contribution of IFN-ß signaling to the observed anabolic phenotype.

Importantly, we also examined the expression of *Gas6*, the primary ligand of Axl, during osteoblast differentiation. Our qPCR analysis revealed that *Gas6* expression progressively increased as osteoblasts mature, in contrast to the declining expression of *Axl*. This divergent temporal pattern suggests that *Gas6* may act in an autocrine or paracrine manner within the osteoblast lineage, potentially allowing differentiated cells to regulate progenitor cells and maintain bone homeostasis. Osteoblast-derived Gas6 has been shown to signal to neighboring cells, including pre-osteoclasts, to promote survival and differentiation via TAM receptors.^24,76,77^ In addition, Gas6 can modulate paracrine interactions with cancer cells in the bone microenvironment.^24,78^ Thus, osteoblast-derived Gas6 may contribute to osteoblast-osteoclast communication, coordinating bone formation and resorption, and may also modulate the anabolic effects of Axl inhibition observed in this study. Although a previous in vitro study suggested that Axl inhibition may impair osteoclast differentiation, these findings were obtained under cell culture conditions.^79^ In contrast, in our in vivo model, treatment with BGB324 did not significantly alter osteoclast parameters, indicating that the predominant skeletal effect is mediated through osteoblast regulation. Future studies using osteoclast-specific *Axl* knockout mice will be required to clarify the role of Axl in osteoclast biology and to further delineate the autocrine and paracrine effects of Gas6 in osteoblasts and osteoclasts under physiological and pathological conditions.

The function of Axl in bone stands in contrast to its known roles in immunity, cancer, and vascular biology, where it promotes survival, migration, and immune suppression.^52,53,80^ Notably, Axl inhibition in macrophages and endothelial cells enhances inflammation.^53,81^ However, in osteoblasts, Axl appears to act primarily as a brake on differentiation. This cell-type-specific role is reminiscent of other RTKs (e.g., FGFRs, PDGFRs) that exhibit divergent functions depending on cellular context.^82,83^ At first glance, the pro-inflammatory consequences of Axl inhibition in immune and endothelial compartments seem counterintuitive to the increased bone mass observed in our healthy mouse model, as chronic inflammation is typically associated with impaired bone formation and enhanced bone resorption.^84,85^ However, our experiments were conducted under baseline physiological conditions, where any modest inflammatory activation resulting from Axl inhibition is unlikely to reach a threshold that negatively impacts bone. Instead, the strong osteoblast-autonomous anabolic effect appears to dominate in the healthy skeletal environment. Nevertheless, it is important to recognize that the outcome may differ in pathological settings characterized by heightened or chronic inflammation. In disease contexts such as rheumatoid arthritis, inflammatory bowel disease-associated bone loss, or metabolic inflammation, Axl inhibition could potentially amplify inflammatory signaling to levels that disrupt osteoblast function or promote osteoclast-mediated bone resorption. Future studies in inflammatory disease models will therefore be essential to determine whether the anabolic effects of Axl inhibition are preserved or attenuated in non-homeostatic conditions.

Therapeutically, the findings have substantial translational relevance. First, we established Axl as a viable osteoanabolic target, potentially expanding the therapeutic landscape for osteoporosis. Second, since BGB324 is already in clinical trials for oncology,^40,41,44^ its repurposing for skeletal disorders could be expedited by leveraging existing safety and pharmacokinetic data. Finally, considering Axl’s role in tumor-bone interactions and bone metastasis,^79,86^ targeting Axl may yield dual benefits in cancer and skeletal disease, though the risks and benefits of such dual targeting will require careful evaluation.

In conclusion, our study reveals Axl as a novel negative regulator of osteoblast differentiation and bone formation, acting through repression of the Erk-Isg15 axis. These findings advance our understanding of bone biology and introduce Axl as a potential therapeutic target for osteoporosis and other conditions characterized by impaired bone formation. Future research should investigate the role of Axl in pathological settings such as aging, estrogen deficiency, and mechanical unloading, and evaluate its long-term impact on bone strength, quality, and fracture resistance.

## Materials and methods

### Animal experimentation

All animal procedures were conducted in accordance with international guidelines for the care and use of laboratory animals (ARRIVE guidelines)^87^ and were approved by the local regulatory authority (Regierungspräsidium Tübingen, License number 1245). Female BALB/cAnNCrl wild-type mice (9- and 11-week-old, Charles River Laboratories) were housed individually under pathogen-free conditions at Ulm University. 129/Sv mice were bred in-house, and newborn pups were used for isolating primary osteoblasts from calvariae. Animals were maintained on a 14-h light and 10-h dark cycle at 23 °C with 55% ± 10% humidity, with *ad libitum* access to food and water.

To inhibit Axl, mice received oral gavage of BGB324 (BerGenBio AS) (50 mg/kg, twice daily, 8 h apart) or vehicle (0.5% (w/v) methylcellulose and 0.1% (w/v) Tween 80 in water), as previously described^88^ (Fig. 5a, Fig. S5a). Body weight was recorded prior to each administration. In the primary cohort, treatment was administered for 14 days to assess early skeletal responses. To evaluate the effects of prolonged Axl inhibition, an independent cohort of mice received the same treatment regimen for 28 days. At the end of the respective treatment periods, mice were euthanized by isoflurane overdose (Baxter, Cat. HDG9623), followed by cardiac blood collection. Skeletal tissues were then harvested for further analysis.

### Primary murine calvarial osteoblast isolation and culture

Primary osteoblasts were isolated from the calvariae of 2- to 5-day-old 129/Sv mice, as previously described.^89^ Briefly, dissected calvariae were incubated in 1 mL of digestion solution containing 0.2% (w/v) Collagenase A (Sigma-Aldrich, Cat. 11088793001) and 0.2% (w/v) Dispase II (Sigma-Aldrich, Cat. 4942078001) at 37 °C with constant shaking (700 r/min) for 10 min. The first digestion supernatant (fraction 1) was discarded. The digestion was then repeated four additional times (fraction 2–5), and the resulting supernatants were pooled into 15 mL tubes containing 500 µL fetal bovine serum (FBS; PAA, Cat. A15-101) to neutralize enzymatic activity.

Cells were pelleted by centrifugation at 1 500 r/min for 5 min at room temperature (RT), resuspended in 3 mL of complete culture medium (α-MEM; GIBCO, Cat. 41061037) supplemented with 10% FBS and 1% penicillin-streptomycin (Thermo Fisher Scientific, Cat. 15140122), and seeded in one well of a six-well plate per calvaria. Cells were maintained at 37 °C in a humidified atmosphere with 5% CO_2_. After 24 h, non-adherent cells were removed by medium replacement. Adherent osteoblasts were cultured for an additional 2–3 days until reaching ~80% confluency.

### Primary osteoblast isolation from long bones and culture

Primary osteoblasts were isolated from long bones of 12-week-old female mice using a previously established protocol.^90,91^ Briefly, femora, tibiae, humeri, and radii/ulnae (two of each per mouse) were harvested under aseptic conditions and cleared of soft tissue. Bones were washed with PBS, and epiphyses were removed. Bone marrow was flushed out by centrifugation at 12 000 r/min for 1 min at RT. Diaphyseal bone fragments were cut into 1–2 mm^2^ pieces and digested with 1 mg/mL Collagenase IV (Sigma-Aldrich, Cat. C5138) in α-MEM (Lonza, Cat. BE12-169F) for 1 h at 37 °C in a shaking water bath. Following two washes with PBS, bone fragments were transferred to 56 cm^2^ culture dishes containing complete growth medium: α-MEM supplemented with 15% fetal bovine serum (FBS; Sigma-Aldrich, Cat. S0615), 1% Penicillin-Streptomycin, and 1% L-glutamine (Thermo Fisher Scientific, Cat. 25030024). Cultures were maintained at 37 °C in a humidified 5% CO_2_ incubator for three weeks. After this period, bone fragments were removed, and adherent cells were harvested by trypsinization. Cells from each mouse were pooled and seeded into two T175 flasks. Experiments were performed when cultures reached 80% confluency.

### Osteoblast differentiation

Primary murine calvarial osteoblasts and long-bone-derived osteoblasts were seeded at a density of 20 000 cell/cm^2^ and allowed to adhere for 48 h. Osteogenic differentiation was initiated by replacing the culture medium with osteogenic induction medium consisting of 100 μg/mL (+)-Sodium L-Ascorbate (Sigma-Aldrich, Cat. A4034) and 5 mmol/L β-glycerophosphate (Sigma-Aldrich, Cat. G5422). Cells were treated with the Axl inhibitor BGB324 (Selleckchem, Cat. S2841) at concentrations of 14 nmol/L and 100 nmol/L in the osteogenic induction medium. Dimethyl sulfoxide (DMSO; Carl Roth, Cat. A994.1) at 0.01% (v/v) was used as a vehicle control. The induction medium and treatments were refreshed every three days.

### siRNA transfection

For siRNA transfection experiments, the following siRNAs were purchased from Horizon Discovery (PerkinElmer): siGENOME Non-Targeting Control siRNA Pool #2 (Cat. D-001206-14-05), siGENOME mouse *Axl* siRNA (Cat. M-040941-01-0005), siGENOME mouse *Tgtp1* siRNA (Cat. M-042868-01-0005), siGENOME mouse *Isg15* siRNA (Cat. M-167630-01-0005), and siGENOME mouse *Zbp1* siRNA (Cat. M-048021-00-0005). Reverse transfection was performed using 0.125% Lipofectamine RNAiMAX transfection reagent (Thermo Fisher Scientific, Cat. 13778075) and a final siRNA concentration of 20 nmol/L, following the procedure described previously.^89^ The sequences of all siRNAs used in this study are listed in Table 2.

**Table 2:** siRNA sequences targeting mouse genes used in this study

Gene	Ensembl ID	siRNA Sequence(s)
Axl	ENSMUSG00000002602	GGAAAGAGGUGAACUGGUA CAAGAUGAAUGGAAAGUUG GGAACUGCAUGCUGAAUGA GGAAGAAGGAGACUCGAUA
Isg15	ENSMUSG00000035692	GGGAACAAGUCCACGAAGA GUACAGAACUGCAGCGAGC AGACUGUAGACACGCUUAA UGGCUGAGCUUCGAGGGAA
Mapk1	ENSMUSG00000063358	UCGAGUUGCUAUCAAGAAA UGAGAGGGCUAAAGUAUAU ACAAGAGGAUUGAAGUUGA UAUACCAAGUCCAUUGAUA
Tgtp1	ENSMUSG00000078922	UCCCAAAGCUGGAAACUAA UCGCAUGGCUUAUUAUUUG UCACCUUGAUUAUGACUUA GAACCAAGAUAGACAGCGA
Zbp1	ENSMUSG00000027514	GGACAGACGUGGAAGAUCU GGAAUGUCAUAGUAAGAGA AUAAGCACCUUCUGAGCUA GUCAAAGGGUGAAGUCAUG
Non-targeting #2	-	UAGCGACUAAACACAUCAA UAAGGCUAUGAAGAGAUAC AUGUAUUGGCCUGUAUUAG AUGAACGUGAAUUGCUCAA

### Cell viability assay

Cell viability was assessed using the PrestoBlue^TM^ Cell Viability Reagent (Thermo Fisher Scientific, Cat. A13261), following the manufacturer’s instructions. Briefly, 275 µL of cell culture medium was removed from each well of a 24-well plate, and 25 µL of PrestoBlue reagent was added directly to the remaining medium. In parallel, a blank control was prepared by mixing the reagent with culture medium at a 1:10 ratio to a final volume of 100 µL in a 96-well plate. All plates were incubated at 37 °C in a humidified incubator with 5% CO_2_ for 30 min. Subsequently, 100 µL aliquots were transferred to a 96-well plate, and absorbance was measured at 570 nm using a Spark Multimode Microplate Reader (TECAN, Cat. 30086376) against the blank control.

### Qualitative and quantitative alkaline phosphatase (Alp) staining

Alkaline phosphatase (Alp) activity was assessed qualitatively and quantitatively using established protocols.^89,92^ For qualitative analysis, cells were washed with PBS and fixed with 4% paraformaldehyde (PFA) for 10 min at RT. Alp staining solution (Sigma-Aldrich, cat. 86R-1KT) was added according to the manufacturer’s instructions, and plates were incubated for 1 h at RT in the dark. Stained cells were imaged using a VHX digital microscope (Keyence, Cat. VHX-7000).

For quantitative analysis, cells were seeded in 24-well plates and cultured in osteogenic induction medium supplemented with 100 μg/mL (+)-Sodium L-Ascorbate and 5 mmol/L β-glycerophosphate. Alp activity was measured using the Amplite^TM^ Colorimetric Alkaline Phosphatase Assay Kit (Biomol, Cat. ABD-11950) following the manufacturer’s protocol. After washing with PBS, cells were incubated with p-nitrophenyl phosphate (pNPP) substrate at 37 °C for 1 h. Absorbance was measured at 405 nm using a Spark Multimode Microplate Reader. Alp activity was normalized to cell viability, and results were expressed as percentage change relative to control (set at 100%).

For ELF 97 staining (Thermo Fisher Scientific, Cat. E6601), primary murine calvarial osteoblasts were seeded in a 384-well plate and induced to differentiate using osteogenic induction medium. Cells were fixed, stained, and analyzed as previously described.^89^

### Qualitative and quantitative Alizarin Red S (ARS) staining

Matrix mineralization of cells was assessed using Alizarin Red S (ARS) staining, as previously described.^89^ For qualitative analysis, cells were washed twice with PBS, fixed with 4% PFA for 10 min at RT, and stained with 1% ARS solution (Sigma-Aldrich, Cat. A5533) for 30 min at RT. After three washes with PBS to remove excess dye, mineralized nodules were imaged using a VHX digital microscope.

For quantitative analysis, ARS-stained cells cultured in 24-well plates were treated with 10% acetic acid following an established protocol.^89^ Plates were incubated at 700 r/min for 30 min at RT. Cells were then scraped, transferred to 1.5 mL microcentrifuge tubes, vortexed briefly, and heated at 85 °C for 10 min. After cooling on ice, samples were centrifuged at 1 500 r/min for 15 min at RT. A 100 µL aliquot of the supernatant was neutralized with 100 µL of 10% ammonium hydroxide. Absorbance was measured at 405 nm using the Spark Multimode Microplate Reader. ARS levels were normalized to cell viability, and results were expressed as percentage change relative to control (set to 100%).

### RNA isolation, cDNA synthesis, and quantitative real-time PCR

Total RNA was extracted from cultured cells using the RNeasy Mini Kit (Qiagen, Cat. 75142) following the manufacturer’s protocol. For RNA isolation from bone, the surrounding soft tissues were carefully removed from the radii/ulnae, and the epiphyses were excised. Bone marrow was flushed out by centrifugation at 12 500 r/min for 1 min at RT. Bones were transferred into tubes containing 1 mL QIAzol lysis reagent (Qiagen, Cat. 79306) and homogenized for 20 s using Miccra D-9 homogenizer (MICCRA, Cat. 090099). The lysates were centrifuged at 14 000 r/min for 10 min at 4 °C to separate the solubilized bone extract from the residual bone fragments. The supernatant was transferred to a new tube, mixed with 200 µL chloroform (VWR, Cat. 22711.290) and centrifuged at 14 000 r/min for 25 min at 4 °C. Total RNA was subsequently purified using the RNeasy Mini kit. For cDNA synthesis, 1 µg of total RNA was reverse transcribed using the Omniscript Reverse Transcriptase kit (Qiagen, Cat. 205113). Quantitative real-time PCR (qRT-PCR) was performed using the QuantStudio 3 Real-Time PCR System (Applied Biosystems). Amplification specificity was verified by melt-curve analysis, and linearity was confirmed using standard curves for each primer pair. Gene expression levels were calculated using the 2^−^^ΔΔCt^ method and normalized to beta-2 microglobulin *(B2m)* or beta actin *(Actb)* as internal reference genes. Primer sequences used in this study are listed in Table 3.

**Table 3:** Mouse oligonucleotide primer sequences used for quantitative real-time PCR (qRT-PCR)

Gene symbol	Forward primer	Reverse primer
Actb	AGAGGGAAATCGTGCGTGAC	CAATAGTGATGACCTGGCCGT
Alpl	GCTGATCATTCCCACGTTTT	CTGGGCCTGGTAGTTGTTGT
Axl	AGCCTTCCTGTGCCCCTA	GAGGTGGGGGTTCACTCA
B2m	ATACGCCTGCAGAGTTAAGCA	TCACATGTCTCGATCCCAGT
Gas6	CCGCGCCTACCAAGTCTTC	CGGGGTCGTTCTCGAACAC
Isg15	GGTGTCCGTGACTAACTCCAT	TGGAAAGGGTAAGACCGTCCT
Mapk1	CCCTCACAAGAGGATTGAAGTT	GGCTCATCACTTGGGTCATAA
Mertk	GTTCTGGCCCCACTGCTA	AGTGCCTCCACTCCAGAGC
Runx2	TGTTCTCTGATCGCCTCAGTG	CCTGGGATCTGTAATCTGACTCT
Sp7	CCCACCCTTCCCTCACTCAT	CCTTGTACCACGAGCCATAGG
Tgtp1	TGCACAGATGGGGATGAATTTC	TCACTGTCGAGAGACTCCTGA
Tyro3	GTGAAGCCCGCAACATAA	CAGTACAGGAATGCAGCAGA
Zbp1	AAGAGTCCCCTGCGATTATTTG	TCTGGATGGCGTTTGAATTGG

### RNA sequencing (RNAseq) and bioinformatic analysis

Total RNA was isolated using the RNeasy Mini Kit, and RNA concentration and integrity were assessed using the 2100 Bioanalyzer with the RNA 6000 Nano Kit (Agilent Technologies). Only RNA samples with an A_260/_A_280_ ratio of 1.9–2.0 and RNA integrity number (RIN) > 9.5 were used for downstream analysis. Library preparation and RNA sequencing were performed by Novogene using the Illumina HiSeq 2500 platform (Illumina). Raw sequencing data were processed with fastp to trim adapter sequences and remove low-quality reads. High-quality reads were aligned to the mouse reference genome (GRCm38.p6, Ensembl) using STAR, and transcript abundance was quantified using featureCounts.

Principal component analysis (PCA) was conducted using the pcaExplorer package^93^ to assess sample variability and clustering. Gene expression was normalized, and differential expression analysis was performed using DESeq2 in RStudio. Differentially expressed genes (DEGs) were defined as those with a log_2_ fold change (log_2_FC) ≥0.5 or ≤−0.5 and a false discovery rate (FDR) < 0.01. DEGs were visualized using volcano plots generated with the EnhancedVolcano package,^94^ and hierarchical clustering was performed using the pheatmap. Gene Ontology (GO) enrichment analysis of significantly up- and downregulated genes was conducted using Metascape.^95^

### Protein isolation, quantification, and western blotting

Total cellular protein was extracted using radioimmunoprecipitation assay (RIPA) lysis buffer and quantified using the Pierce BCA protein assay kit (Cat. 23225; Thermo Fisher Scientific) according to the manufacturer’s instructions. Equal amounts of protein (25 µg per sample) were resolved by sodium dodecyl sulfate-polyacrylamide gel electrophoresis (SDS-PAGE) and transferred onto nitrocellulose membranes for immunoblotting, as previously described.^89^ Membranes were incubated with primary antibodies against Axl (R&D Systems, Cat. AF854), ISG15 (Cell Signaling Technology, Cat. 2473), Phospho-p44/42 MAPK (Erk1/2) (Thr202/Tyr204; Cell Signaling Technology, Cat. 9101), total p44/42 MAPK (Erk1/2; Cell Signaling Technology, Cat. 9102), and alkaline phosphatase/ALPL (R&D Systems, Cat. AF2910). α-Tubulin (Cell Signaling Technology, Cat. 2144) and β-Actin (13E5; Cell Signaling Technology, Cat. 4970) were used as loading controls. Bound antibodies were detected using HRP-conjugated secondary antibodies (Anti-mouse IgG (Cell Signaling Technology, cat. 7076), Anti-rabbit IgG (Cell Signaling Technology, Cat. 7074), and goat anti-mouse IgG (Sigma-Aldrich, Cat. AP308P)). Bands were visualized using the Immobilon Forte Western HRP substrate (Millipore, Cat. WBLUF0500) and imaged by chemiluminescence. The band intensity of western blots was quantified using ImageJ.^96^

### Microcomputed tomography (µCT) analysis

High-resolution microcomputed tomography (µCT) was performed on femurs and vertebrae using a Skyscan 1176 scanner (Bruker) at an isotropic voxel size of 9 µm. Scans were acquired using an X-ray source set at 50 kV and 200 µA with a 0.5 mm aluminum filter and a rotation step of 1°. Image reconstruction was performed using NRecon software (v1.7.5.9, Bruker), and datasets were reoriented using DataViewer (v1.6.0.0, Bruker). For trabecular and cortical bone analysis, regions of interest (ROIs) were defined starting approximately 0.45 mm and 4.05 mm from the distal growth plate, extending proximally by 2.70 mm and 0.90 mm, respectively. For the vertebral trabecular analysis, the ROI encompassed the body of the fifth lumbar vertebra (L5). For the skull, the ROI was defined 1.8 mm up from the lambdoid suture, and a measurement of 0.08 mm was recorded. Structural parameters were quantified using CTAn software (v1.20.8.0, Bruker), and representative three-dimensional reconstructions were generated with CTvox software (v3.3.1, Bruker). All µCT analyses were conducted in accordance with the guidelines of the American Society for Bone and Mineral Research (ASBMR).^97^

### Bone histomorphometry

For static histomorphometric analysis, femurs were harvested, fixed in 4% PFA for 48 h at 4 °C, decalcified in 15% EDTA for 10 days, embedded in paraffin, and sectioned at 5 µm thickness. Sections were deparaffinized and stained for tartrate-resistant acid phosphatase (TRAP) by incubating in TRAP staining solution for 1 h at RT, followed by hematoxylin counterstaining for 3 min. Slides were mounted using Fluoromount^TM^ Aqueous Mounting Medium (Sigma-Aldrich, Cat. F4680). Osteoblast, osteoclast, and osteocyte parameters were quantified on TRAP-stained sections using the OsteoMeasure high-resolution digital video analysis system (OsteoMetrics). For dynamic histomorphometry, mice received intraperitoneal injections of calcein (20 mg/kg: Sigma-Aldrich, Cat. C0875) at 9 and 2 days prior to sacrifice, as previously described.^31^ Femurs were dissected, cleaned of soft tissue, fixed in 4% PFA, and embedded in methyl methacrylate (MMA) according to established protocols.^31,98^ Histomorphometric analysis was performed on longitudinal 5 µm sections using the OsteoMeasure system, following the guidelines of the ASBMR.^97^ Due to technical limitations during preparation of undecalcified sections, dynamic histomorphometric analyses were performed on slightly reduced sample numbers (typically *n* = 5, with one group *n* = 4).

### Immunohistochemistry

Paraffin-embedded sections were deparaffinized through xylene and graded ethanol, followed by antigen retrieval in 10 mmol/L citrate buffer (pH 6.0) at 95 °C for 20 min. Endogenous peroxidase activity was blocked with 3% H₂O₂, and non-specific binding was minimized using 5% BSA in TBS for 1 h. Sections were incubated overnight at 4 °C with a sheep anti-mouse Dmp1 antibody (R&D Systems, Cat. AF4386, 1:200) or sheep IgG as a negative control. After washing, biotinylated rabbit anti-sheep secondary antibody (Vector, Cat. BA-6000, 1:250) and Avidin-Biotin Complex (Vector, Cat. PK-6100) were applied. Signal was developed using NovaRED (Vector, Cat. SK-4800), counterstained with hematoxylin, blued in running water, dehydrated through ethanol and xylene, and mounted with Vitro-Clud (Langenbrinck, Cat. 04-0001).

### Single mRNA in situ hybridization

Single-molecule mRNA in situ hybridization was performed using the RNAscope Multiplex Fluorescent Reagent Kit according to the manufacturer’s instructions (ACDBio, Cat. 323110) and as previously described.^99^ Femurs from 12-week-old male mice were fixed in 4% PFA, paraffin-embedded, and sectioned at 5 µm onto SuperFrost™ Plus glass slides. Sections were incubated at 60 °C for 1 h, deparaffinized in xylene, dehydrated in 100% ethanol, and treated with 30% hydrogen peroxide for 10 min at RT. After protease treatment (ACDBio, Cat. 322381) for 20 min at 40 °C, sections were hybridized with the RNAscope probe targeting mouse *Axl* (mm-*Axl*, ACDBio, Cat. 450931) for 2 h at 40 °C. Signal amplification was performed sequentially using Amplification Reagents 1–3 according to the manufacturer’s protocol, followed by HRP incubation and fluorophore development using TSA reagent (1:1 000).

For combined immunofluorescence staining, sections were blocked in 3% BSA and 0.3% Triton X-100 in PBS and incubated with primary antibodies against Sp7 (rabbit anti-Sp7, Abcam, Cat. ab209484), CD90.2 (rat anti-mouse CD90.2, Invitrogen, Cat. 14-0902-82), and CD44 (rat anti-mouse CD44, BD Pharmingen, Cat. 561860), followed by Alexa Fluor-conjugated secondary antibodies (donkey anti-rabbit Alexa Fluor™ 488, Thermo Fisher Scientific, Cat. A-21206; goat anti-mouse Alexa Fluor™ 750, Thermo Fisher Scientific, Cat. A-21037). Sections were mounted using ProLong™ antifade mounting medium (Invitrogen, Cat. P36934). Fluorescence images were acquired using an epifluorescence microscope (Keyence, Cat. BZ-X810).

### Enzyme-linked immunosorbent assays (ELISAs)

Whole blood was collected into ethylenediaminetetraacetic acid (EDTA)-coated tubes, incubated at RT for 15 min, and centrifuged at 2 000 r/min for 10 min at RT to obtain plasma. Plasma concentrations of Procollagen Type I N-Terminal Propeptide (PINP) (Immunodiagnostic Systems, Cat. AC-33F1) and C-terminal telopeptide of type I collagen (CTX-I) (Immunodiagnostic Systems, Cat. AC-07F1) were measured using ELISA kits according to the manufacturer’s protocols.

### Statistical analysis

The data are displayed as scattered dot plots, with the mean and standard deviation. Statistical differences between groups were determined using one-way ANOVA followed by Tukey’s post hoc test. A *P*-value less than 0.05 was considered statistically significant.

## Supplementary information

Supplementary Material

## Data Availability

The data supporting the findings of this study are available from the corresponding author upon reasonable request.
