# Supplementary material for: Inhibition of AXL receptor tyrosine kinase increases osteoblast function and bone mass
Source: Bone Res. 2026 Jul 6;14:71. doi: 10.1038/s41413-026-00554-0 (PMC13338172; doi:10.1038/s41413-026-00554-0)
Supplement: Supplementary file 1 — Supplementary Material [file 41413_2026_554_MOESM1_ESM.docx]

Title:

Inhibition of AXL receptor tyrosine kinase increases osteoblast function and bone mass

Running Title:

*Axl suppression increases bone mass*

Mubashir Ahmad^1,2,*^, Christoph Kölbl^2^, Irfana Jan^1,3^, Ann-Kristin Dorn^1^, Burak Özkan^4^, Florian Olde Heuvel^4^, Dilay Yilmaz^1^, Alessa Wagner^1^, Maja Vujic Spasic^1^, Benjamin Thilo Krüger^2^, Sooyeon Lee^1^, Francesco Roselli^4,5^, Anita Ignatius^2,*^, Jan Tuckermann^1,6,*^

^1^Institute of Comparative Molecular Endocrinology and Physiology, Ulm University, 89081 Ulm, Germany.

^2^Institute of Orthopedic Research and Biomechanics, Ulm University Medical Center, 89081 Ulm, Germany.

^3^Institute of Immunology, Ulm University Medical Center, 89081 Ulm, Germany.

^4^Department of Neurology, Ulm University, 89081 Ulm, Germany.

^5^German Center for Neurodegenerative Disease (DZNE)-Ulm, 89081 Ulm, Germany.

^6^German Center for Child and Adolescent Health (DZKJ), Partner Site Ulm, 89081 Ulm, Germany.

*These authors contributed equally.

**Author for Correspondence:**

Prof. Dr. Jan Tuckermann; Institute of Comparative Molecular Endocrinology and Physiology, Ulm University, Helmholtzstrasse 8/1, 89081 Ulm, Germany.; Tel.: +49 731 5032600.

Email: [jan.tuckermann@uni-ulm.de](mailto:jan.tuckermann@uni-ulm.de)

Prof. Dr. Anita Ignatius; Institute of Orthopedic Research and Biomechanics, Ulm University Medical Center, Helmholtzstrasse 14, 89081 Ulm, Germany.; Tel.: +49 731 50055338.

Email: [anita.ignatius@uni-ulm.de](mailto:anita.ignatius@uni-ulm.de)

Dr. Mubashir Ahmad; Institute of Orthopedic Research and Biomechanics, Ulm University Medical Center, Helmholtzstrasse 14, 89081 Ulm, Germany.; Tel.: +49 731 50055338.

Email: [ahmad.mubashir@uni-ulm.de](mailto:ahmad.mubashir@uni-ulm.de)

## Authors contributions

M.A., A.I., and J.T. conceived and designed the experiments. M.A., C.K., I.J., A.-K.D., B.Ö., F.-O.H., D.Y., A.W., M.-V.S., B.-T.K., S.L., and F.R. performed the experiments. M.A., C.K., and I.J. analyzed the data. M.A., A.I., and J.T. interpreted the data and wrote the original draft. All authors reviewed and approved the final version of the manuscript.

# Supplementary Information


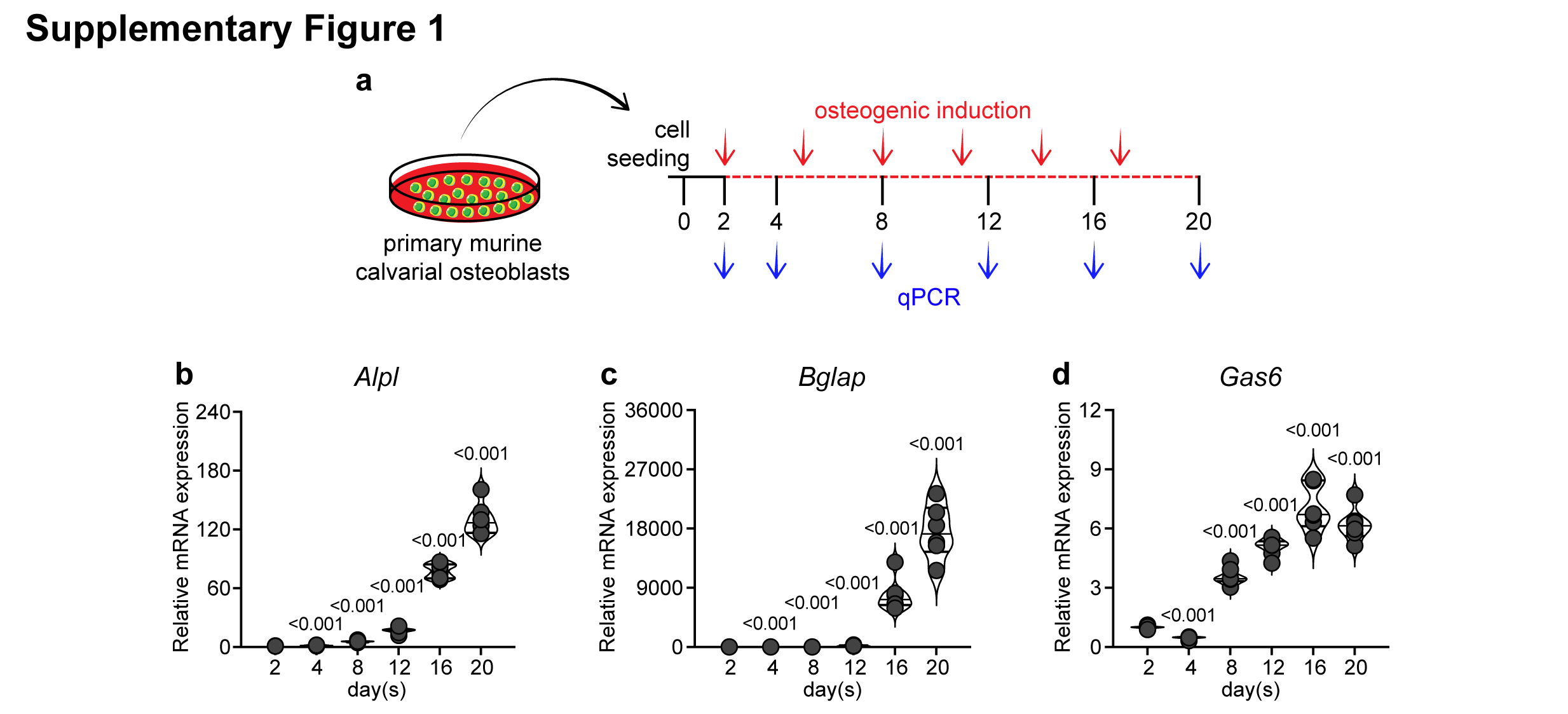


**Supplementary Fig. 1 | Osteoblast marker gene expression during the course of osteoblast differentiation.** **(a)** Schematic, and **(b-d)** temporal analysis of *Alpl*, *Bglap*, and *Gas6* mRNA expression during osteoblast differentiation in primary murine calvarial osteoblasts, assessed by qRT-PCR (n=6). Data are presented as aligned dot plots showing the mean and standard deviation. Statistical comparisons between time points were performed using one-way ANOVA followed by Tukey’s post hoc test.


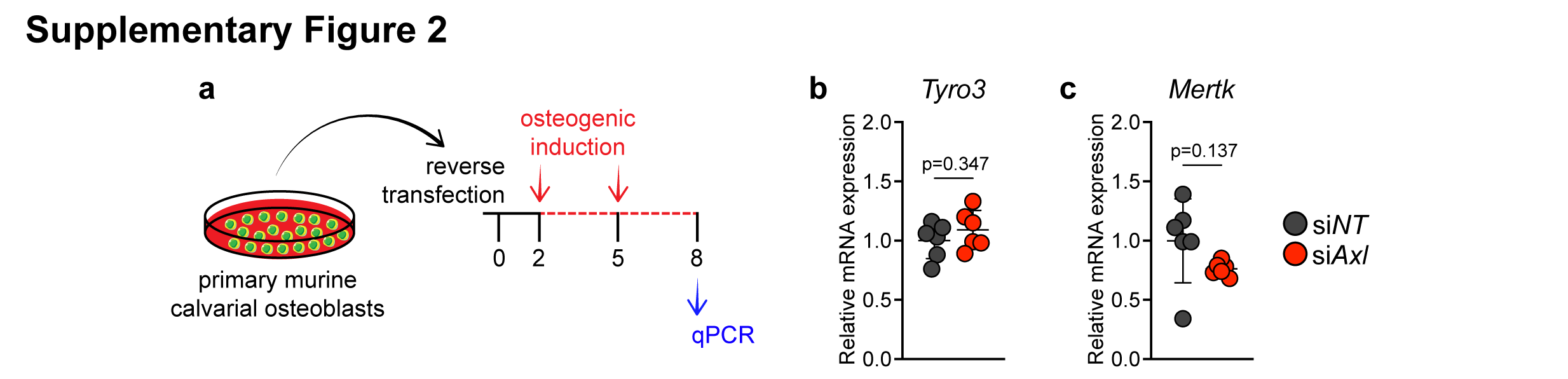


**Supplementary Fig. 2 | *Axl* knockdown does not alter *Tyro3* and *Mertk* mRNA expression in primary murine calvarial osteoblasts. (a)** Schematic overview of siRNA-mediated knockdown of *Axl* in primary murine calvarial osteoblasts. **(b,c)** qRT-PCR analysis of *Tyro3* and *Mertk* mRNA expression at day 8 post-transfection in si*NT-* or si*Axl-*transfected cells (n=6). Data are presented as scatter dot plots with mean and standard deviation. Statistical significance between two groups was determined using one-way ANOVA followed by Tukey’s post hoc test.


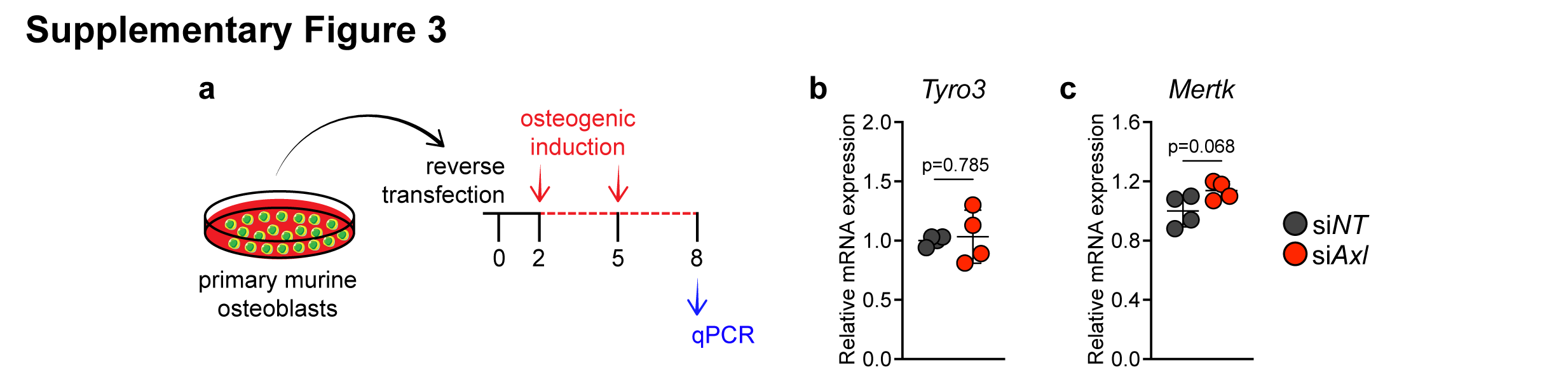


**Supplementary Fig. 3 | *Axl* knockdown does not alter *Tyro3* and *Mertk* mRNA expression in primary murine osteoblasts. (a)** Schematic overview of siRNA-mediated knockdown of *Axl* in primary murine osteoblasts. **(b,c)** qRT-PCR analysis of *Tyro3* and *Mertk* mRNA expression at day 8 post-transfection in si*NT-* or si*Axl-*transfected cells (n=6). Data are presented as scatter dot plots with mean and standard deviation. Statistical significance between two groups was determined using one-way ANOVA followed by Tukey’s post hoc test.


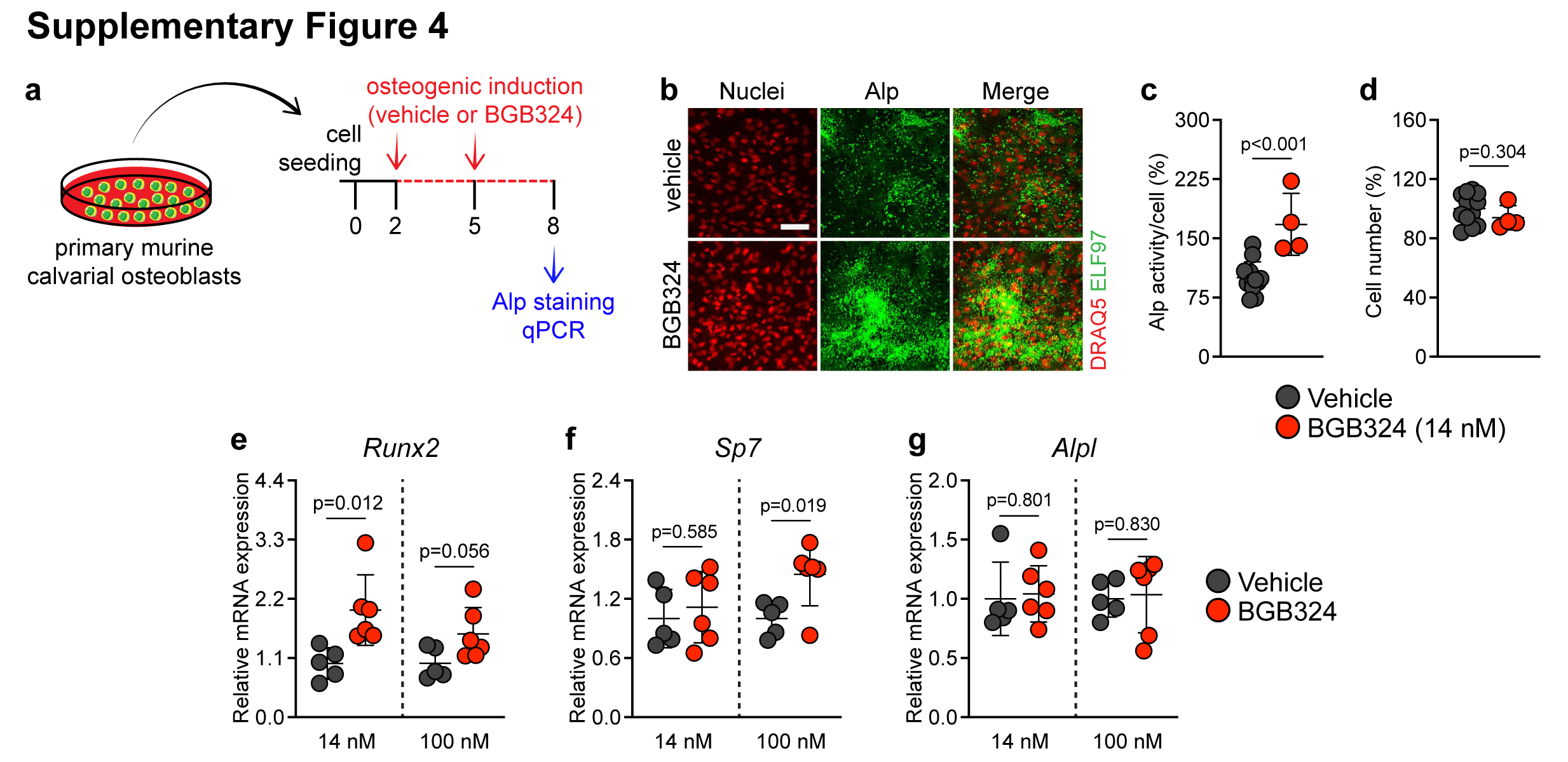


**Supplementary Fig. 4 | Pharmacological inhibition of Axl with BGB324 *in vitro* promotes osteoblast differentiation in primary murine calvarial osteoblasts. (a)** Schematic overview of vehicle (DMSO) or BGB324 (14 nmol.L^-1^ or 100 nmol.L^-1^) treatment in primary murine calvarial osteoblasts. **(b)** Representative fluorescence images of primary murine calvarial osteoblasts treated with vehicle or BGB324 at day 8 post-seeding, showing nuclear staining with DRAQ5 (red) and Alp activity with ELF 97 (green). Scale bar, 100 µm. **(c,d)** Quantification of cellular Alp activity and cell numbers in primary murine calvarial osteoblasts treated with vehicle or BGB324 at day 8 post-seeding (n=4-12). **(e-g)** Expression of osteoblast-specific marker genes *(Runx2, Sp7,* and *Alpl)* measured by qRT-PCR in vehicle- or BGB324-treated cells at day 8 post-seeding (n=5-6). Data are presented as scatter dot plots with mean and standard deviation. Statistical significance between two groups was determined using one-way ANOVA followed by Tukey’s post hoc test.


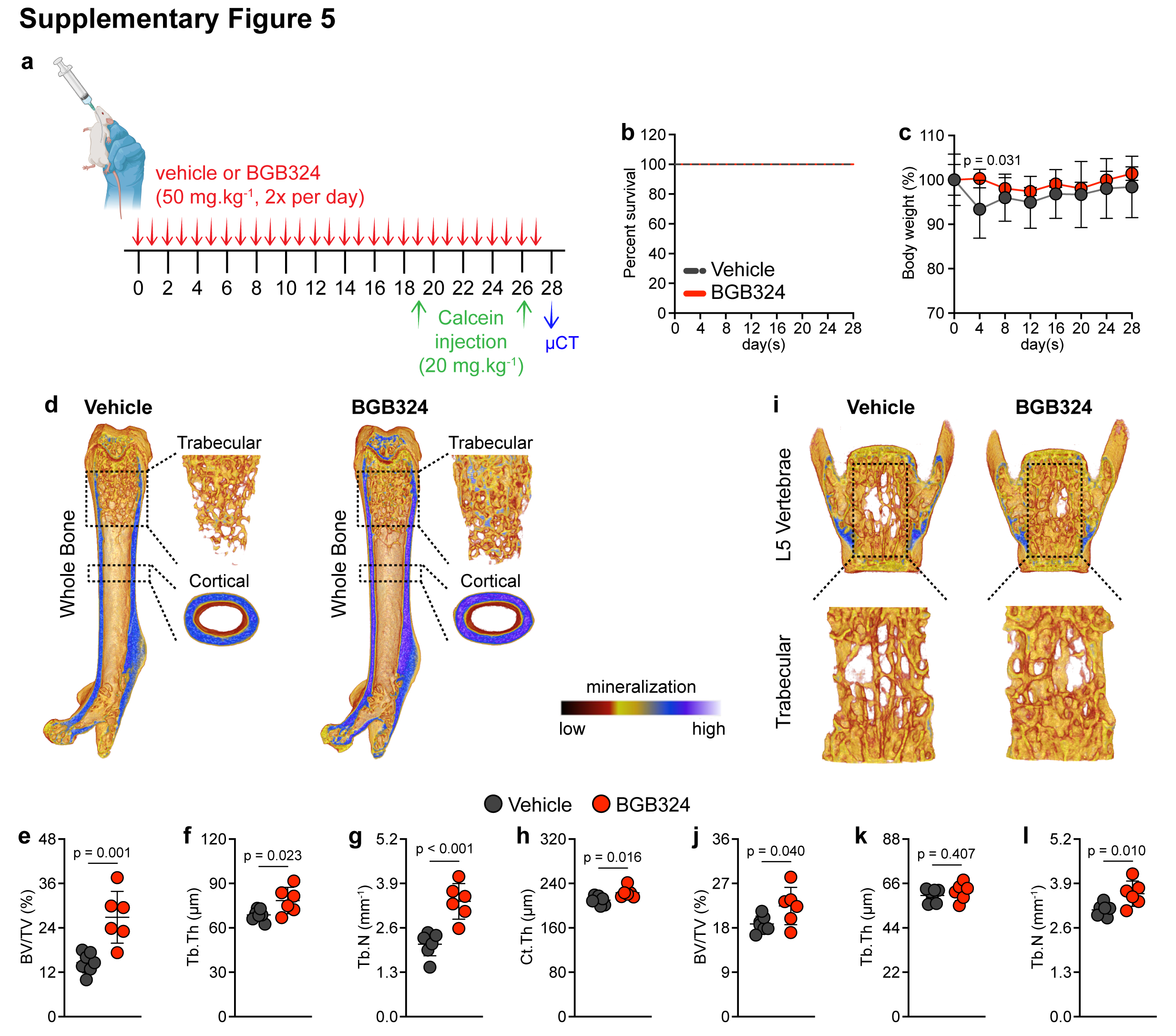


**Supplementary Fig. 5 | Pharmacological inhibition of Axl with BGB324 increases bone mass *in vivo*. (a)** Experimental design for treatment of 9-week-old female BALB/c mice with vehicle (0.5% (w/v) methylcellulose and 0.1% (w/v) Tween 80 in water) or BGB324 (50 mg.kg^-1^, administered by oral gavage twice daily, 8 hours apart for four weeks). **(b,c)** Percent survival and body weight of mice treated with vehicle or BGB324 (n=6-7). **(d)** Representative microcomputed tomography (µCT) images of whole femur, trabecular bone, and cortical bone from vehicle- or BGB324-treated mice. **(e-h)** Quantification of femoral bone parameters: bone volume fraction (BV/TV, %), trabecular thickness (Tb.Th, µm), trabecular number (Tb.N, mm^-1^), and cortical thickness (Ct.Th, µm) (n=6-7). **(i)** Representative µCT images of whole vertebra and trabecular bone from vehicle- or BGB324-treated mice. **(j-l)** Quantification of vertebral trabecular bone parameters: BV/TV (%), Tb.Th (µm), and Tb.N (mm^-1^) (n=6-7). Data are presented as scatter dot plots with mean and standard deviation. Statistical significance between two groups was determined using one-way ANOVA followed by Tukey’s post hoc test.


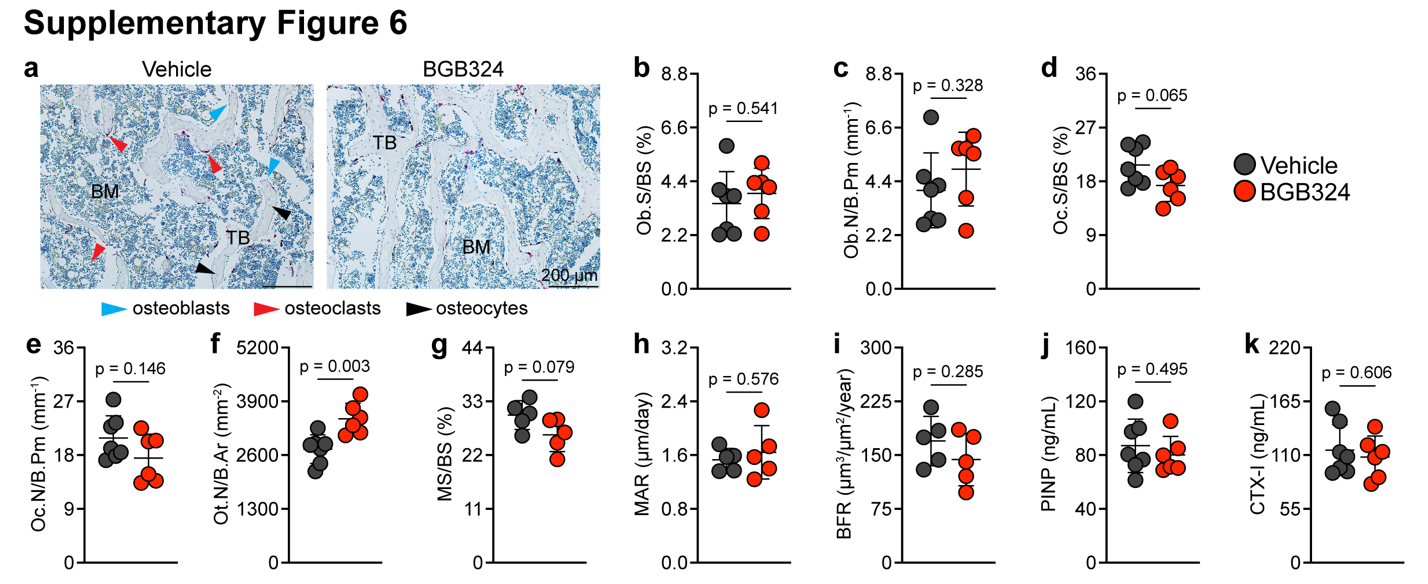


**Supplementary Fig. 6 | Pharmacological inhibition of Axl with BGB324 promotes maturation of osteoblasts into osteocytes *in vivo*. (a)** Representative tartrate-resistant acid phosphatase (TRAP) staining for femoral trabecular bone sections from vehicle- or BGB324-treated mice, showing osteoblasts (blue arrows), osteoclasts (red arrows), and osteocytes (black arrows) (scale bar: 200 µm). **(b-f)** Quantification of histomorphometric parameters from femoral trabecular bone: osteoblast surface per bone surface (Ob.S/BS, %), osteoblast number per bone perimeter (Ob.N/B.Pm, mm^-1^), osteoclast surface per bone surface (Oc.S/BS, %), osteoclast number per bone perimeter (Oc.N/B.Pm, mm^-1^), and osteocyte number per bone area (Ot.N/B.Ar, mm^-2^) (n=6-7). **(g-i)** Dynamic bone histomorphometry of femurs from vehicle- or BGB324-treated mice showing mineralizing surface per bone surface (MS/BS, %), mineral apposition rate (MAR, µm/day), and bone formation rate (BFR, µm^3^/µm^2^/year) (n=5). **(j,k)** Plasma levels of bone formation and resorption markers: procollagen type I N-terminal propeptide (PINP, ng/mL) and C-terminal telopeptide of type I collagen **(**CTX-I, ng/mL) (n=6-7). Data are presented as scatter dot plots with mean and standard deviation. Statistical significance between two groups was determined using one-way ANOVA followed by Tukey’s post hoc test.


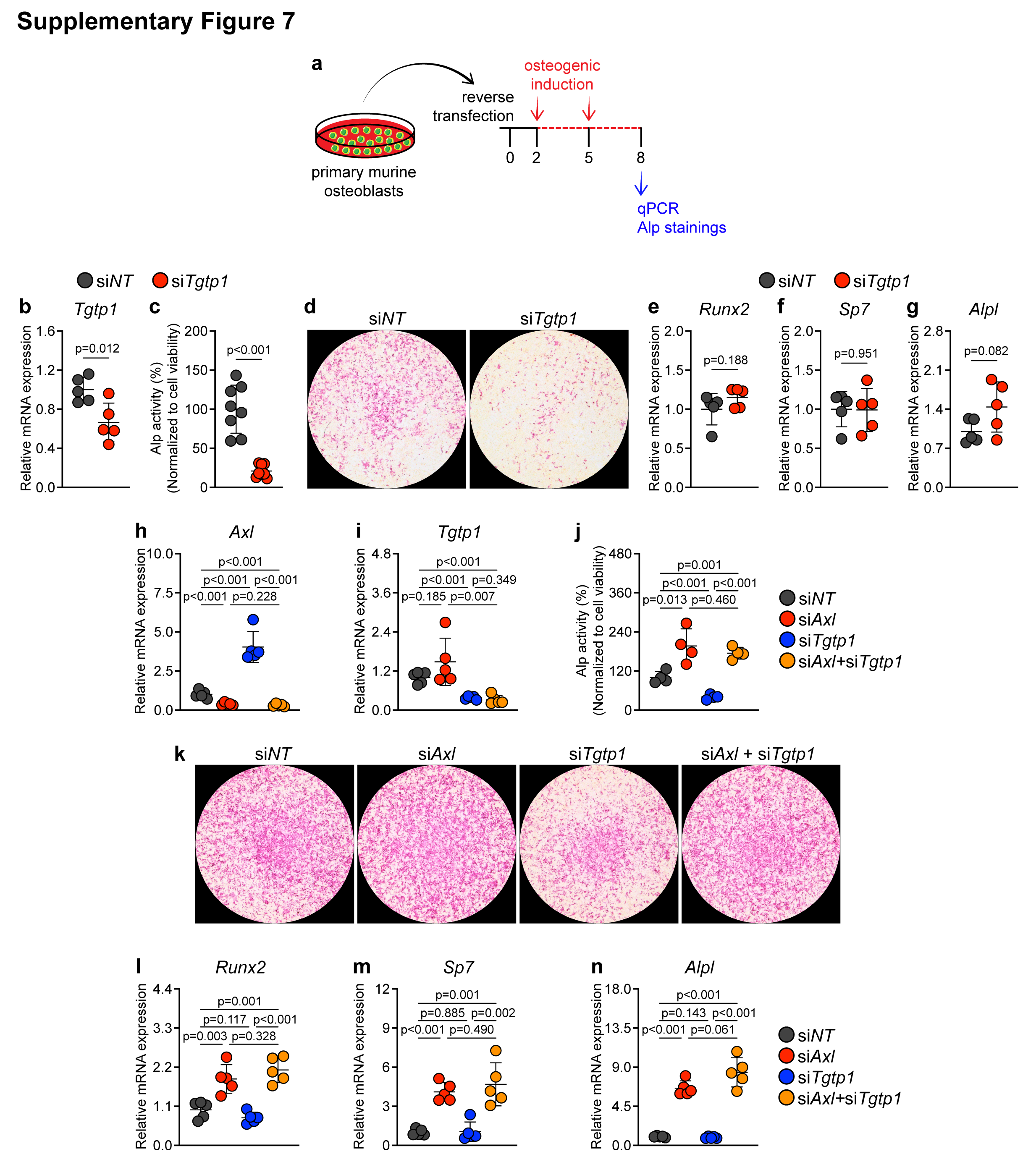


**Supplementary Fig. 7 | *Tgtp1* knockdown impairs osteoblast differentiation in primary murine osteoblasts. (a)** Schematic overview of siRNA-mediated knockdown of *Tgtp1* in primary murine osteoblasts. **(b)** *Tgtp1* mRNA expression measured by qRT-PCR at day 8 post-transfection (n=5). **(c,d)** Quantitative and qualitative Alp staining in cells transfected with si*NT* or si*Tgtp1* at day 8 post-transfection (n=8). **(e-g)** qRT-PCR analysis of osteoblast-specific marker genes *(Runx2, Sp7,* and *Alpl)* in si*NT*- or si*Tgtp1*-transfected cells at day 8 post-transfection (n=5). **(h,i)** *Axl* and *Tgtp1* mRNA expression measured by qRT-PCR at day 8 post-transfection (n=5). **(j,k)** Quantitative (n=6) and qualitative (n=6) Alp staining in cells transfected with si*NT*, si*Axl*, si*Tgtp1*, or co-transfection of si*Axl* and si*Tgtp1* at day 8 post-transfection. **(l-n)** Expression of osteoblast-specific marker genes *(Runx2, Sp7,* and *Alpl)* quantified by qRT-PCR in cells transfected with si*NT*, si*Axl*, si*Tgtp1*, or co-transfection of si*Axl* and si*Tgtp1* at day 8 post-transfection (n=5). Data are presented as scatter dot plots with mean and standard deviation. Statistical significance between two groups was determined using one-way ANOVA followed by Tukey’s post hoc test.


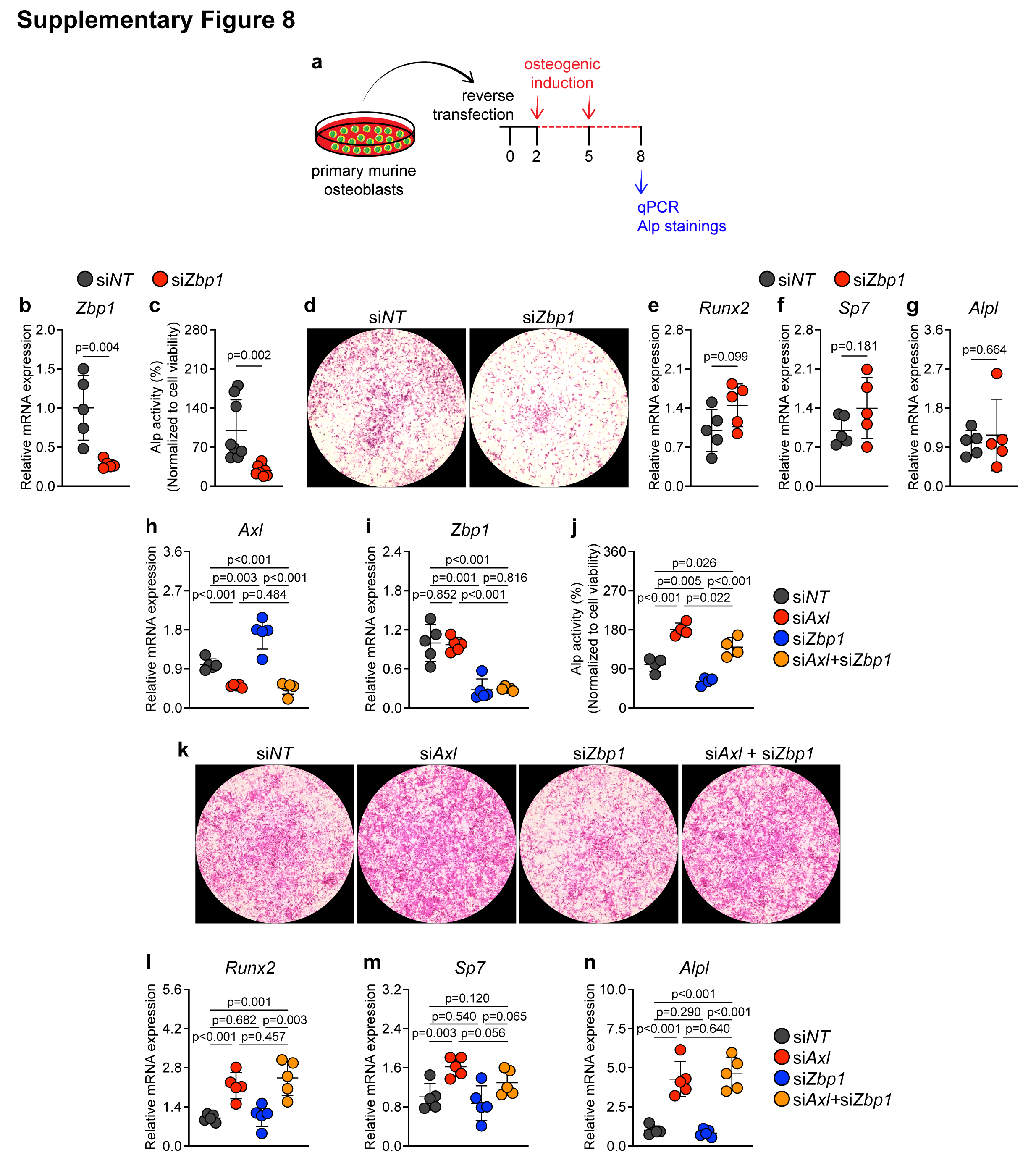


**Supplementary Fig. 8 | *Zbp1* knockdown impairs osteoblast differentiation in primary murine osteoblasts. (a)** Schematic overview of siRNA-mediated knockdown of *Zbp1* in primary murine osteoblasts. **(b)** *Zbp1* mRNA expression measured by qRT-PCR at day 8 post-transfection (n=5). **(c,d)** Quantitative and qualitative Alp staining in cells transfected with si*NT* or si*Zbp1* at day 8 post-transfection (n=8). **(e-g)** qRT-PCR analysis of osteoblast-specific marker genes *(Runx2, Sp7,* and *Alpl)* in si*NT*- or si*Zbp1*-transfected cells at day 8 post-transfection (n=5). **(h,i)** *Axl* and *Zbp1* mRNA expression measured by qRT-PCR at day 8 post-transfection (n=5). **(j,k)** Quantitative (n=6) and qualitative (n=6) Alp staining in cells transfected with si*NT*, si*Axl*, si*Zbp1*, or co-transfection of si*Axl* and si*Zbp1* at day 8 post-transfection. **(l-n)** Expression of osteoblast-specific marker genes *(Runx2, Sp7,* and *Alpl)* quantified by qRT-PCR in cells transfected with si*NT*, si*Axl*, si*Zbp1*, or co-transfection of si*Axl* and si*Zbp1* at day 8 post-transfection (n=5). Data are presented as scatter dot plots with mean and standard deviation. Statistical significance between two groups was determined using one-way ANOVA followed by Tukey’s post hoc test.
